# Solubility, speciation and thermodynamics of Fe in reducing aqueous KCl solutions

**DOI:** 10.1039/d5ra07073b

**Published:** 2025-11-25

**Authors:** Paul Q. Fürst, Neşe Çevirim-Papaioannou, Xavier Gaona, Krassimir Garbev, Thomas Roth, Sven Hagemann, Marcus Altmaier, Horst Geckeis

**Affiliations:** a Institute for Nuclear Waste Disposal, Karlsruhe Institute of Technology Karlsruhe Germany p.fuerst@kit.edu xavier.gaona@kit.edu; b Institute for Technical Chemistry, Karlsruhe Institute of Technology Karlsruhe Germany; c Gesellschaft für Anlagen- und Reaktorsicherheit Braunschweig Germany

## Abstract

The solubility of two well-characterized Fe(ii) solid phases (*i.e.*, Fe(OH)_2_(cr) and Fe_2_(OH)_3_Cl(cr)) were investigated in batch undersaturation solubility experiments conducted over a wide range of pH_m_ (7.5 ≤ pH_m_ ≤ 10.5) and ionic strength (0.01 M ≤ *I* ≤ 4.0 M KCl). Solid phase characterization was carried out using XRD including Rietveld analysis, providing key insights into their phase composition and crystallite size. Chemical, thermodynamic and SIT activity models were derived for the system Fe^2+^–K^+^–H^+^–Cl^−^–OH^−^–H_2_O(l) on the basis of the comprehensive experimental dataset. Solubility constants determined in this work 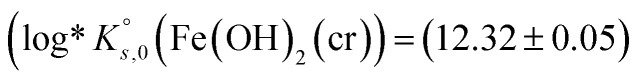 and 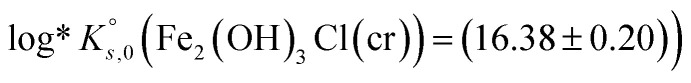 contribute to improving the description of Fe chemistry under very reducing conditions, and can be implemented in thermodynamic databases and geochemical calculations of relevance, *e.g.* in the context of nuclear waste disposal.

## Abstract 2

Iron corrosion phases influence chemical conditions in several anthropogenic applications. New experimental data and thermodynamic models derived in this work allow accurate predictions of Fe behavior under relevant environmental conditions.

## Graphical abstract

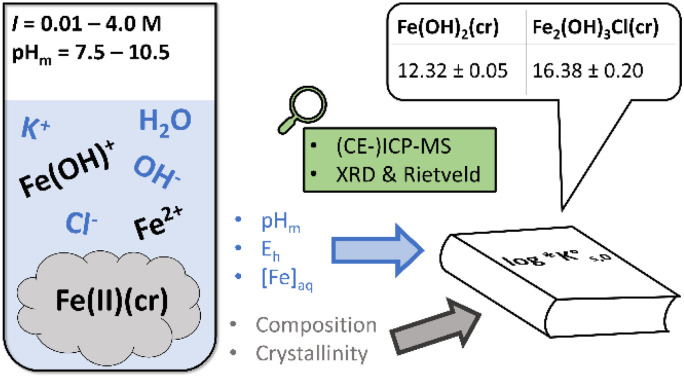

## Introduction

As a common element in the Earth's crust, iron (Fe) plays a key role in countless environmental systems. A proper understanding of its chemistry accordingly provides essential insights to numerous processes occurring in the geosphere. Knowledge on aquatic chemistry of iron is also of high relevance in the context of underground repositories for nuclear waste disposal, where metallic iron, particularly as cast iron and steel, is extensively used as container material, construction material and, in cases of low and intermediate level radioactive waste, also as a waste constituent. In the post-closure phase of the repository, the anoxic corrosion of iron components with the corresponding formation of hydrogen is expected to play a major role in determining the reducing geochemical conditions in the nearfield of such repositories, thus strongly influencing the retention behavior of radionuclides.

Ziemniak *et al.* investigated the solubility of Fe_3_O_4_(cr) (magnetite) under very reducing conditions defined by the presence of H_2_ (*p* = 1 atm). Based on their solubility data and solid phase characterization, the authors concluded that Fe(OH)_2_(cr) (“white rust”) is expected to form as the Fe(ii) solubility determining species up to a transformation temperature of 389 K.^1^ However, note that Fe(OH)_2_(cr) can only have a stability field at all if 
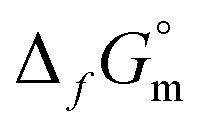
 is negative for the reaction:^2^

1 Fe_3_O_4_(cr) + Fe(cr) + 4H_2_O(l) ⇌ 4Fe(OH)_2_(cr)

Considering the thermodynamic data selected for Fe(cr) and Fe_3_O_4_(cr) in the context of the Thermochemical Database project of the Nuclear Energy Agency (NEA-TDB),^2,3^ the condition above is only fulfilled for 
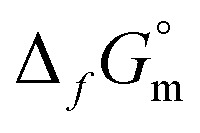
 (Fe(OH)_2_(cr)) < −(490.3 ± 0.4) kJ mol^−1^ for Fe(OH)_2_(cr) at 298.15 K and consequently a 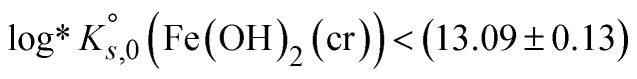. These values are calculated on the basis of bulk materials with well-ordered crystalline structures, which may not be those controlling the solubility of Fe under repository-relevant conditions. Indeed, several studies investigating the solubility of Fe(OH)_2_(cr)^1,4–7^ reported 
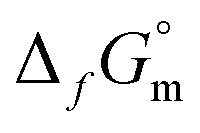
 below this upper limit, thus supporting the stabilization of this solid phase under very reducing conditions. This underpins the relevance of Fe(ii) solid phases in the anoxic corrosion of metallic iron, and by extension in the redox couples controlling the redox in underground repositories, *e.g.*, Fe(cr)/Fe(OH)_2_(cr) or Fe(OH)_2_(cr)/Fe_3_O_4_(cr). Most of these studies were critically reviewed in Volumes 13a and 13b of the NEA-TDB focusing on the chemical thermodynamics of iron (2013,^2^ 2020^3^). Following the strict selection criteria within the NEA-TDB, thermodynamic data for Fe(OH)_2_(cr) were not selected, mainly due to insufficient characterization of the solid phases used in the solubility studies, as well as the lack of systematic investigations. On the basis of data proposed by Chase *et al.* in the NIST-JANAF thermochemical tables^8^ from calorimetric experiments and formerly reviewed by Chivot *et al.*,^9^ the ThermoChimie database^10^ selected a value of 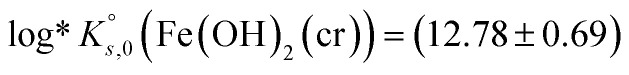 (see Table 1). Thermodynamic data selected in the PSI/Nagra Chemical Thermodynamic Database^11^ for this solid phase followed the discussion provided by Brown & Ekberg,^12^ who calculated 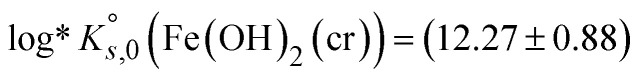 based on a similar dataset as discussed within the NEA-TDB.^2^ Both reference databases acknowledge the uncertainties associated to this solid phase with the large errors associated with the corresponding solubility product.

**Table 1:** Equilibrium constants reported and selected in the literature for solubility, hydrolysis and chloro complexation of Fe(ii) containing solid phases and aqueous species

Reaction	log*K°/log*β°	Ref.
Fe(OH)_2_(cr) + 2H^+^ ⇌ Fe^2+^ + 2H_2_O	(12.78 ± 0.69)	aTC 12a^10^
	(12.27 ± 0.88)	Brown & Ekberg^12^
	(12.26 ± 0.88)	PSI/Nagra TDB^11^
Fe_4_(OH)_8_Cl(cr) + 8H^+^ + e^−^ ⇌ 4Fe^2+^ + Cl^−^ + 8H_2_O	(41.80 ± 2.79)	TC 12a^10^
β-Fe_2_(OH)_3_Cl(s) + 3H^+^ ⇌ 2Fe^2+^ + Cl^−^ + 3H_2_O	(17.2 ± 0.2)	NEA-TDB^3^
Fe^2+^ + H_2_O ⇌ FeOH^+^ + H^+^	−(8.8 ± 0.5)	Bruno *et al.* (2018)^16^
	−(9.1 ± 0.4)	aNEA-TDB^2^
	−(9.25 ± 0.24)	TC 12a^10^
	−(9.43 ± 0.10)	PSI/Nagra TDB^11^/Brown & Ekberg^12^
Fe^2+^ + 2H_2_O ⇌ Fe(OH)_2_(aq) + 2H^+^	−(20.4 ± 0.7)	Bruno *et al.* (2018)^16^
	−(20.84 ± 1.74)	aTC 12a^10^
	−(20.52 ± 0.08)	PSI/Nagra TDB^11^/Brown & Ekberg^12^
Fe^2+^ + Cl^−^ ⇌ FeCl^+^	−(1.0 ± 0.8)	aNEA-TDB^2^ (recommended value)
Fe^3+^ + 2H_2_O ⇌ Fe(OH)_2_^+^ + 2H^+^	−(4.8 ± 0.4)	aNEA-TDB^2^
Fe^3+^ + 3H_2_O ⇌ Fe(OH)_3_(aq) + 3H^+^	−(12.56 ± 0.50)	aTC 12a^10^
Fe^3+^ + 4H_2_O ⇌ Fe(OH)_4_^−^ + 4H^+^	−(21.60 ± 0.50)	aTC 12a^10^

a Data used for calculation of solubility curve and Pourbaix diagram for comparison with the experimental data collected in the present work.

Fe_2_(OH)_3_Cl(cr) can likely be formed as corrosion product of Fe(cr) in chloride-rich anoxic brines and naturally occurs in three polymorphs: α-Fe_2_(OH)_3_Cl (no mineral name, hexagonal),^13^ β-Fe_2_(OH)_3_Cl parahibbingite (trigonal/hexa-rhombohedral) and γ-Fe_2_(OH)_3_Cl hibbingite (orthorhombic).^14^ Nemer *et al.*^6,15^ investigated the solubility of hibbingite in NaCl and Na_2_SO_4_ brines and determined a value for log*K° (Fe_2_(OH)_3_Cl(cr)) of (17.2 ± 0.2) for the reaction given in Table 1, which was later selected by the NEA-TDB.^3^

The present study aims at developing comprehensive chemical, thermodynamic and SIT activity models for the system Fe^2+^–K^+^–H^+^–Cl^−^–OH^−^–H_2_O(l) on the basis of systematic undersaturation solubility experiments combined with accurate solid phase characterization. The solubility of well-defined Fe(ii) solid phases, specifically Fe(OH)_2_(cr) and Fe_2_(OH)_3_Cl(cr) is investigated in KCl systems with concentrations ranging from 0.01 to 4.0 M, under near-neutral to alkaline conditions. While repository-relevant brines are inherently complex multicomponent systems, investigations in pure KCl solutions yield fundamental thermodynamic and speciation data for iron. Such baseline information is indispensable for developing robust models of iron behavior under repository conditions.

## Experimental

### Chemicals

All experiments were conducted at *T* = (25 ± 2) °C in a glovebox under argon atmosphere (<0.1 ppm O_2_) to minimize the impact of atmospheric oxygen and carbon dioxide on the systems. All samples and solutions were prepared with ultra-pure water that was purified using a Milli-Q apparatus (Millipore, 18.2 MΩ, 25 ± 2 °C) and purged with argon gas for several hours to minimize the fraction of dissolved O_2_(g) and CO_2_(g). NaOH Titrisol©, KOH Titrisol© and HCl Titrisol© standard solutions, as well as potassium chloride EMSURE® (KCl), were purchased from Merck. 2% HNO_3_ solution (prepared from 60% HNO_3_ Ultrapur, Merck) was used to dilute samples prior to ICP-MS (Inductively Coupled Plasma Mass Spectrometry) analysis. Since commercially sourced solid iron(ii) chloride (hydrate) was observed to contain significant traces of iron(iii), 1.0 M of FeCl_2_ was obtained as turquoise solution by dissolving iron powder (Fe(0), purity > 99.5%, Merck) in 2 M HCl solution under constant argon flow. The suspension was constantly stirred and heated up to 80 °C in order to accelerate iron dissolution. The redox purity of the resulting solution was confirmed by capillary electrophoresis coupled to ICP-MS (CE-ICP-MS) (>95%). For CE-ICP-MS, 1,10-phenanthroline monohydrate (phen, ≥ 99.0%, Carl Roth) and 1,2-cyclohexylenedinitrilotetraacetic acid monohydrate (CDTA, ≥ 99.0%, Merck) were used to complex Fe(ii) and Fe(iii) and stabilize the redox state. The method is described in more detail below.

### Solid phase preparation and characterization

Fe(OH)_2_(cr) solid phases were precipitated by slow addition of a 4 M NaOH/KOH solution to a 1.0 M acidic FeCl_2_ solution until no further precipitate was formed and the pH of the supernatant remained alkaline (pH ≈ 13). The obtained solid was separated from the supernatant and stored as suspension in 0.1 M KOH solution or water (no difference on the solid phase was observed with different aging conditions within ∼1–4 months). A similar approach was followed for the synthesis of Fe_2_(OH)_3_Cl(cr) (hibbingite): namely, 1.0 M aqueous FeCl_2_ solution was titrated with a 4 M KOH solution until the pH remained around neutral (pH ∼7), resulting in a [Cl^−^] to [OH^−^] ratio >> 2.5 to avoid co-precipitation of Fe(OH)_2_(cr).^17^ For the same reason, the precipitate remained in the FeCl_2_-containing supernatant for storage. The Fe_2_(OH)_3_Cl(cr) solid was aged for at least two months and washed one to three times with the respective matrix solution before conducting the solubility experiments.

The synthesized solid phases were characterized before and after the solubility experiments using X-ray diffraction (XRD), in order to identify the structure and crystallite size of the solid phase/s controlling the solubility and observe potential changes that may have occurred in the course of the solubility experiment. An aliquot (∼1–2 mg) from the starting solid phases and selected solubility samples after attaining equilibrium conditions were washed with water (1–3 times) to remove any residues originating from the matrix solution. Due to the high redox sensitivity of dry Fe(ii) solid phases, samples were promptly measured after drying them on an air-tight dome sample holder (silicone, Bruker) for around 20 minutes under Ar atmosphere. In addition, a second container was used to transport the XRD dome from the glovebox to the XRD apparatus, minimizing the risk of atmospheric oxygen diffusion into the dome during transit. Commonly, XRD measurements were started within 10 minutes following the drying of the solid phases and lasted for around 5–10 minutes to avoid oxidation during measurement. XRD data were collected using a Bruker D8 Advance diffractometer with a Cu-Kα X-ray source and a LYNXEYE XE-T detector within 2° ≤ 2*θ* ≤ 80°, with a step size of 0.012° and accumulation times of 0.05–0.1 s per step.

The XRD powder patterns were qualitatively evaluated with HighscorePlus v.5 (Malvern-Panalytical). The Rietveld refinements with quantitative analyses were performed with Topas7 (Bruker AXS) using the fundamental parameters approach. For the synthetic Fe(OH)_2_ (“white rust”) series the structures from ICSD 107289, ICSD 53992 and ICSD 117105 were tested as initial model for Fe(OH)_2_. In addition, structures for fougerite (Fe^II^_4_Fe^III^_2_Cl_2−*x*_(OH)_12+*x*_, green rust chloride (GR-Cl), COD ID: 9011596), akaganeite (Fe_8_O_8_(OH)_8_Cl_1.35_, “β-FeOOH”, ICSD 96830) and magnetite (Fe_3_O_4_) were used for quantitative analysis. For the hibbingite series, the following structure data were used as starting parameters: parahibbingite (β-Fe_2_(OH)_3_Cl, orthorhombic, ICSD 12960), hibbingite (γ-Fe_2_(OH)_3_Cl, trigonal, ICSD 139200), rokuehnite (FeCl_2_·2H_2_O, ICSD 15597), akaganeite (Fe_8_O_8_(OH)_8_Cl_1.35_, ICSD 96830), fougerite (Fe^II^_4_Fe^III^_2_Cl_2−*x*_(OH)_12+*x*_, COD ID: 9011596).

The size of the coherent scattering domains (LVol-IB, Table 2) was determined by the double Voigt approach (Topas 7 reference manual, Bruker AXS), which is a volume-weighted calculation based on integral breadth convolution of Gauss and Lorentz components for “crystal size”.

Phase composition of solid phases used initially and after equilibration according to Rietveld refinements. * Crystal size after double Voigt approach. **Sum of β-Fe_2_(OH)_3_Cl and γ-Fe_2_(OH)_3_Cl

**Table:** 

Sample Fe(OH)_2_(cr)	Phase composition in wt% (e.s.d.)					Fe(OH)_2_
	Amakinite Fe(OH)_2_	Sylvite KCl	Fougerite Fe_4_^2+^Fe_2_^3+^Cl_2−*x*_(OH)_12+*x*_	Akaganeite Fe_8_O_8_(OH)_8_Cl_1.35_	Magnetite Fe_3_O_4_	Crystal size LVol-IB * (nm)
Fe(OH)_2_(cr)	100					132(2)nm
0.01 M KCl, pH_m_ 8.0	99.5(4)	0.5(4)				239(9)nm
0.01 M KCl, pH_m_ 10.1	95.8(4)				4.2(4)	177(4)nm
0.1 M KCl, pH_m_ 8.7	100					265(16)nm
0.1 M KCl, pH_m_ 10.0	97.6(5)	2.4(5)				254(10)nm
0.5 M KCl, pH_m_ 8.2	97.0(9)	1.0(2)	2.0(9)			161(3)nm
0.5 M KCl, pH_m_ 9.5	92.5(7)	7.5(7)				194(6)nm
1.0 M KCl, pH_m_ 8.1	90.7(8)			9.3(8)		218(8)nm
1.0 M KCl, pH_m_ 9.5	100					320(20)nm
2.0 M KCl, pH_m_ 8.1	95.0(7)		5.0(7)			173(4)nm
2.0 M KCl, pH_m_ 9.5	100					239(8)nm
4.0 M KCl, pH_m_ 9.0	89(2)	7(2)	3.5(7)			165(5)nm
4.0 M KCl, pH_m_ 9.4	95(1)	5(1)				219(9)nm

**Table:** 

Sample Fe_2_(OH)_3_Cl(cr)	Phase composition in wt% (e.s.d.)						Fe_2_(OH)_3_Cl
	Parahibbingite β-Fe_2_(OH)_3_Cl	Hibbingite γ-Fe_2_(OH)_3_Cl	Sylvite KCl	Fougerite (Fe^2+,3+^)_6_Cl_2−*x*_(OH)_12+*x*_	Akaganeite Fe_8_O_8_(OH)_8_Cl_1.35_	Rokuehnite FeCl_2_(H_2_O)_2_	Crystal size LVol-IB * (nm)
Fe_2_(OH)_3_Cl (cr)	96.7(5)	3.3(5)					32.2(5)
0.5 M KCl, pH_m_ 7.6	90.0(8)	5.9(6)		3.9(6)		0.2(2)	31.8(5)
0.5 M KCl, pH_m_ 8.5	76.3(3)	5.0(3)	0.7(4)	6.0(7)	12(1)		32(1)
1.0 M KCl, pH_m_ 7.7	88(1)	1.8(9)	0.09(7)	5.9(6)	3.3(4)	0.5(4)	30.7(6)
1.0 M KCl, pH_m_ 8.4	79(1)	3(1)	0.9(3)	8(1)	8.7(7)	0.7(5)	31.3(9)
2.0 M KCl, pH_m_ 8.1	87.2(9)	2.3(7)	0.4(1)	6.9(6)	3.1(4)		31.7(6)
2.0 M KCl, pH_m_ 9.4	93(1)	2.0(1)		5(2)			33(1)
4.0 M KCl, pH_m_ 8.1	90.6(9)	2.6(8)	1.1(2)	4.8(6)	0.8(2)		31.1(6)
4.0 M KCl, pH_m_ 9.7	95(2)	2(1)		3(1)			31.3(10)

**Table:** 

Sample mixed	Phase composition in wt% (e.s.d.)				Crystal size LVol-IB * (nm)	
	Hibbingite^**^ Fe_2_(OH)_3_Cl	Amakinite Fe(OH)_2_	Fougerite (Fe^2+,3+^)_6_Cl_2−*x*_(OH)_12+*x*_	Akaganeite Fe_8_O_8_(OH)_8_Cl_1.35_	Fe_2_(OH)_3_Cl	Fe(OH)_2_
0.5 M KCl, pH_m_ 7.9		96.0(6)	2.1(5)	1.9(2)		121(3)
1.0 M KCl, pH_m_ 8.0	41.3(9)	53.9(9)	3.6(6)	1.2(2)	32(1)	113(3)
2.0 M KCl, pH_m_ 8.5	50.6(9)	44.3(9)	4.6(7)	0.4(2)	29.8(9)	76(3)
4.0 M KCl, pH_m_ 9.1	70(3)	27(2)	1.4(2)	1.6(4)	32(2)	110(9)

### Undersaturation solubility experiments

Undersaturation solubility experiments were conducted in 15 ml screw cap tubes (Sarstedt) by adding approximately 10 mg of the solid phases to 10 ml matrix solution after washing the solid with the respective background solution or Milli-Q water. In total, 52 batch samples were prepared in the following systems: (i) six series in 0.01, 0.1, 0.5, 1.0, 2.0 and 4.0 M KCl/KOH solutions (at 7.6 ≤ pH_m_ ≤ 10.2) were prepared with Fe(OH)_2_(cr), (ii) four series in 0.5, 1.0, 2.0 and 4.0 M KCl/KOH solutions (at 7.6 ≤ pH_m_ ≤ 9.6) were investigated using Fe_2_(OH)_3_Cl(cr) as solid phase, (iii) four samples including a 1 : 1 mixture of both Fe(OH)_2_(cr) and Fe_2_(OH)_3_Cl(cr) solid phases were prepared in 0.5, 1.0, 2.0 and 4.0 M KCl solutions (pH_m_ ≈ 8–9). The matrix solutions were prepared by dissolving KCl solid in oxygen-free water to obtain solutions with KCl concentrations of 0.01, 0.1, 0.5, 1.0, 2.0 and 4.0 M. To maintain the ionic strength constant, HCl or KOH solutions with the same ionic strength as the background electrolyte were used to adjust the pH_m_. The samples were then monitored over the course of up to 226 days, including regular measurements of pH_m_, *E*_h_ and the aqueous iron concentration. Equilibrium conditions were assumed after repeated measurements with constant iron concentrations and pH_m_.

### pH and *E*_h_ measurements

All pH measurements were conducted using a combination glass electrode (type Orion Ross, Thermo Scientific) calibrated with standard pH buffers (pH 8–12, Merck). The pH values are reported as pH_m_, which defines the negative decadic logarithm of the molal H^+^ concentration in accordance with pH_m_ = −log *m*(H^+^) = pH_exp_ + *A*_m_. The experimentally determined pH_exp_ values were corrected using the empirical *A*_m_ parameter that includes both the activity coefficient of the proton as well as the difference in liquid junction potential of the electrode for a given background electrolyte concentration, as described by Altmaier *et al.*^18^*A*_m_ values for the KCl system were adapted from the literature.^19^

To investigate the redox potential, *E*_h_ measurements were conducted using a combined Pt and Ag/AgCl reference electrode (Metrohm). Measurements were performed in suspension, including constant mixing of the samples, usually around 15 minutes until stable *E*_h_ readings were obtained. The measured potentials were corrected for the potential of the Ag/AgCl inner reference electrode at *T* = (25 ± 2) °C with 3 M KCl as electrolyte (+207 mV) in order to calculate *E*_h_ values. *E*_h_ was converted to pe, being the negative decadic logarithm of the electron activity, according to *E*_h_ = −*R*T·ln(10)·*F*^−1^·pe, with *R* being the ideal gas constant and the Faraday constant *F*.

### Aqueous Fe concentration *via* (CE-)ICP-MS

The concentration of iron was monitored at regular time intervals. Phase separation was achieved by ultrafiltration with 10 kDa filters (pore size ≈ 2–3 nm, Nanosep, Pall Life Science). The resulting solutions were acidified and diluted (dilution factor 3.8–10^4^) with 2% HNO_3_ to reach a total salt concentration below 200 mg l^−1^ and analyzed using a multi-quadrupole ICP-MS apparatus (NexION 5000, PerkinElmer or 8900 ICP-QQQ, Agilent). The effective detection limit of the technique ranged between 10^−5.5^–10^−7.5^ mol l^−1^ Fe, depending upon the dilution factors applied for different KCl concentrations.

Capillary electrophoresis coupled to ICP-MS (CE-ICP-MS) was used for selected samples to further investigate the redox state of iron in solution. A method previously reported by Pozdniakova *et al.*^20^ for the determination of Fe by CE-UV/Vis was adapted for CE-ICP-MS^21^ using an Agilent 7100 CE system (Agilent Technologies), which was coupled to the mass spectrometer (NexION 2000B, PerkinElmer) *via* a Mira Mist CE Interface (Burgener Research). To an aliquot (20–100 µl) of the sample solution, a 50 mM solution of 1,10-phenanthroline monohydrate (∼100 µl) was added to quantitatively complex Fe(ii) present in the sample as Fe(ii)-phen. Subsequently, a saturated solution of 1,2-cyclohexylenedinitrilotetraacetic acid monohydrate (∼100 µl) was added to quantitatively complex the Fe(iii) sample content as Fe(iii)-CDTA. Thus, the Fe(ii)/Fe(iii) redox couple was stabilized as complexes with different charges for separation *via* CE. If necessary, the complex solution was diluted to an Fe concentration suitable for detection with CE-ICP-MS of *ca.* 3 × 10^−5^ M with ultra-pure water. The resulting solution was measured with CE-ICP-MS (electrolyte: 100 mM borate, pH 9.2; separation potential: 30 kV) and the peak areas of the Fe(ii)(phen)_3_^2+^ and Fe(iii)(CDTA)^−^ signals at their respective time in the time-resolved mass spectrum were used to estimate a lower limit of the sample Fe(ii)/Fe(iii) ratio.

### Chemical, thermodynamic and SIT activity models

Solubility data in combination with solid phase characterization are used to derive the chemical (*i.e.,* the set of chemical reactions defining the aqueous system) and thermodynamic models. The use of a background electrolyte with sufficiently high concentration ensures that ionic strength and thus activity factors are reasonably constant throughout the measurements. For the description of the solubility or complex formation in aqueous systems with *I* > 0, activity coefficients *γ*_*j*_ are required in addition to thermodynamic equilibrium constants at *I*_m_ = 0. The model developed within this study is based on the Specific Ion Interaction Theory (SIT)^22^ derived from the Brønsted–Guggenheim–Scatchard specific ion interaction method, which provides an extension of the classical Debye–Hückel theory and is also the theoretical model used in the NEA-TDB. The SIT introduces empirical ion interaction coefficients *ε*_*jk*_ for each ion *j* present in the solution accounting for short-range, non-electrostatic interactions, considering also differences between ions of the same charge but different size. An activity coefficient for an ion *j* with charge *z*_*j*_ can be calculated according to eqn (2)2
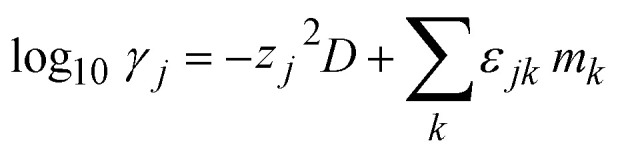


with *m*_*k*_ being the molal concentration of *k* and the temperature, pressure and ionic strength 
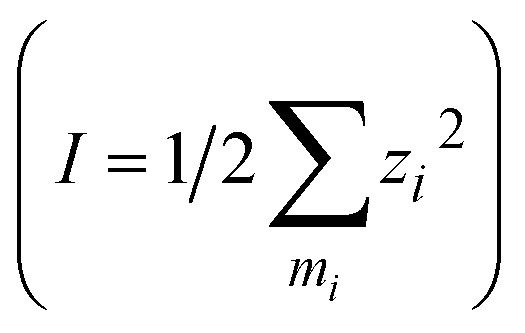
 dependent Debye–Hückel term *D*. For *T* = 25 °C and *p* = 1 bar, *D* is shown in eqn (3).3
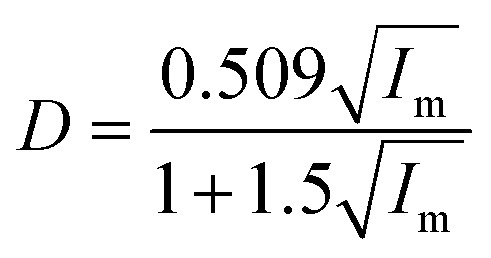


According to SIT, ions with the same positive or negative charge are relatively far apart and short-range interactions between them are therefore negligible. The same is generally considered for uncharged species. In consequence, ion interaction coefficients for those species are assumed to be zero for simplification. In addition, ion interaction coefficients are not independent from the ionic strength and can only be assumed as fairly constant for 1 : 1, 1 : 2 or 2 : 1 electrolytes for molalities up to 3.5 m. However, this limit was safely extended to higher ionic strength by several studies,^23–27^ especially for 1 : 1 electrolytes.

## Results & discussion

### Solid phase characterization

**Fig. 1:**
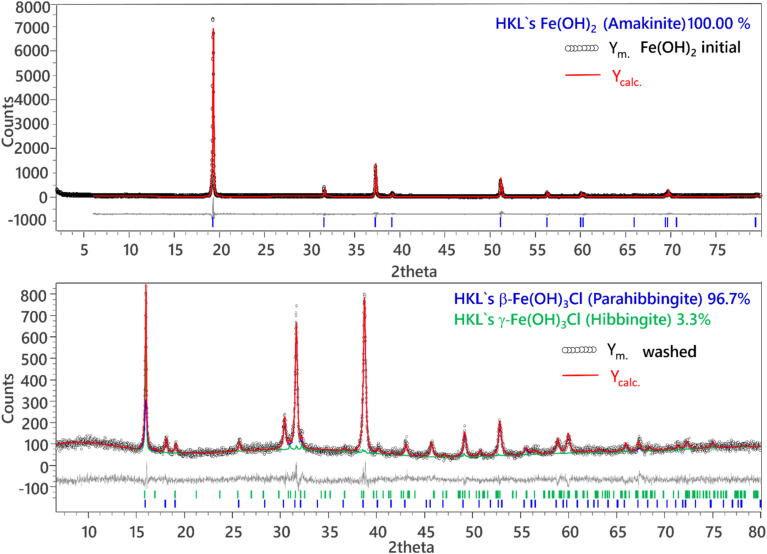
Top: Rietveld plot of Fe(OH)_2_(cr), bottom: Rietveld plot of washed hibbingite.

**Fig. 2:**
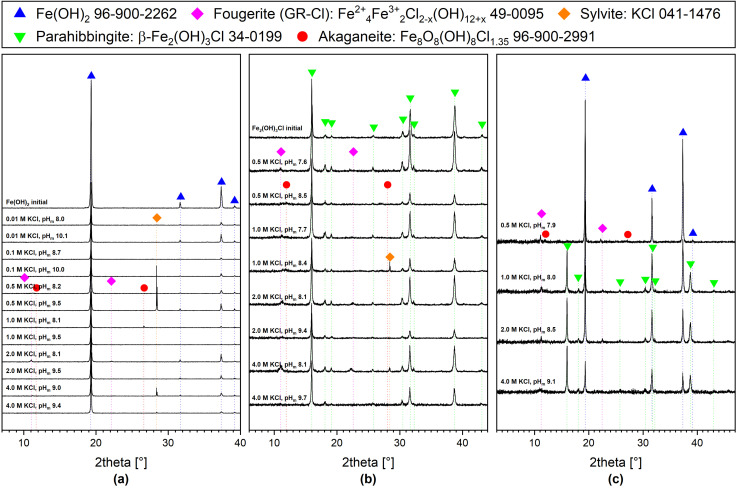
XRD patterns of (a) synthesized Fe(OH)_2_(cr) and selected samples from solubility experiments where this phase was used as initial solid, (b) synthesized Fe_2_(OH)_3_Cl(cr) and selected samples from solubility experiments where this phase was used as initial solid, (c) samples from solubility experiments where a mixture of Fe(OH)_2_(cr) and Fe_2_(OH)_3_Cl(cr) was used as initial solid phase. Main reflections of the phases identified by Rietveld are marked with symbols.

XRD data collected for the starting solid phases are shown in Fig. 1 and 2. Both solid phases are in excellent agreement with reference data reported for Fe(OH)_2_(cr) and Fe_2_(OH)_3_Cl(cr) in the ICDD database^28^ or the Crystallography Open Database^29^ (COD). Fig. 2 also presents the XRD patterns of the selected samples from each ionic strength investigated after attaining equilibrium conditions. Results from Quantitative X-ray Diffraction (QXRD) analyses with the Rietveld method are shown in Table 2 along with calculated sizes of coherent scattering domains of the main phases. Additional information, like unit cell parameters, criteria of fit and corresponding Rietveld plots, is shown in Fig. SI 1 and Fig. SI 2. Fig. 1 (top) shows the Rietveld plot of the initial sample Fe(OH)_2_(cr). There are three structural proposals for Fe(OH)_2_(cr) based on space group *P*3̄*m*1 (164), all considering Fe^2+^ on *x* = 0, *y* = 0, *z* = 0 position (1a) and O^2−^ on *x* = 2/3, *y* = 1/3 (2d). Whereas Natta and Casazza (1927)^30^ and Wyckoff (1963)^31^ propose for *z* = 0.27 and 0.25, respectively, Parise *et al.*^32^ give *z* = 0.2213(2). In addition, they refined the position of H. Therefore, we refined once again the structure proposed by Parise *et al.*^32^ and confirmed their results (Table SI 1). The data were further used for refinement of all samples of this series.

In the experiments conducted with Fe(OH)_2_(cr), the solid phases remained largely unchanged; however, in systems with high KCl concentrations (*I* ≥ 0.5 M KCl) at near-neutral pH conditions (pH_m_ ≈ 8), a feature at 2*θ* = 11, in addition to the features of Fe(OH)_2_(cr), was observed. These features hint towards the presence of traces of green rust chloride (GR-Cl), constituting approximately 4–7% of the solid phase, based on Rietveld analysis. Indeed, the formation of GR-Cl is known to be favored at near-neutral conditions in the presence of Cl^−^.^33,34^ The transformation of this small portion of the total solid phase is expected to have a negligible impact on the overall solubility behavior as the solubility of Fe(iii) at these pH values is expected to be several orders of magnitude lower.^35^

In the hibbingite experiments, 96.7 wt% of the initial material is present as β-Fe_2_(OH)_3_Cl (parahibbingite), with a minor fraction of γ-Fe_2_(OH)_3_Cl and free from impurities. Therefore, this sample will be further referred to as Fe_2_(OH)_3_Cl(cr). The Rietveld plot is shown in Fig. 1 (bottom). Partial transformation of Fe_2_(OH)_3_Cl(cr) into Fe(OH)_2_(cr) during the experiments was observed for several samples above a certain pH_m_. To ensure that the presented model is only based on the solubility behavior of Fe_2_(OH)_3_Cl(cr), those samples were excluded from the model. The pH_m_ above which transformation was observed increased with increasing ionic strength. This behavior hints towards the interplay between Fe(OH)_2_(cr) and Fe_2_(OH)_3_Cl(cr) as a function of pH_m_ and chloride concentration. This is further confirmed in the mix experiments initially containing both solid phases at pH_m_ ≈ 8, for which Fe(OH)_2_(cr) is the only phase remaining after attaining equilibrium conditions in 0.5 M KCl, and the systematic increase in the fraction of Fe_2_(OH)_3_Cl(cr) with increasing KCl concentration, up to 70% in 4.0 M KCl (see Table 2). Moreover, mixed systems showed a stronger tendency to form traces of Fe(iii) containing solids such as GR-Cl and to a lesser extent akaganeite compared to pure solids. This is possibly due to the combination of high chloride concentration and the structural similarity between Fe(OH)_2_(cr) and GR-Cl. Since the formation of GR-Cl traces under these conditions was observed to occur within minutes after drying the samples on the sample holder, even under Ar atmosphere, it is likely that GR-Cl observed in the XRD was mainly formed during the sample preparation and measurement. Nevertheless, the presence of small traces of GR-Cl formed in the course of the solubility experiment cannot be completely excluded.

### Solubility measurements

#### Fe(OH)_2_(cr)

**Fig. 3:**
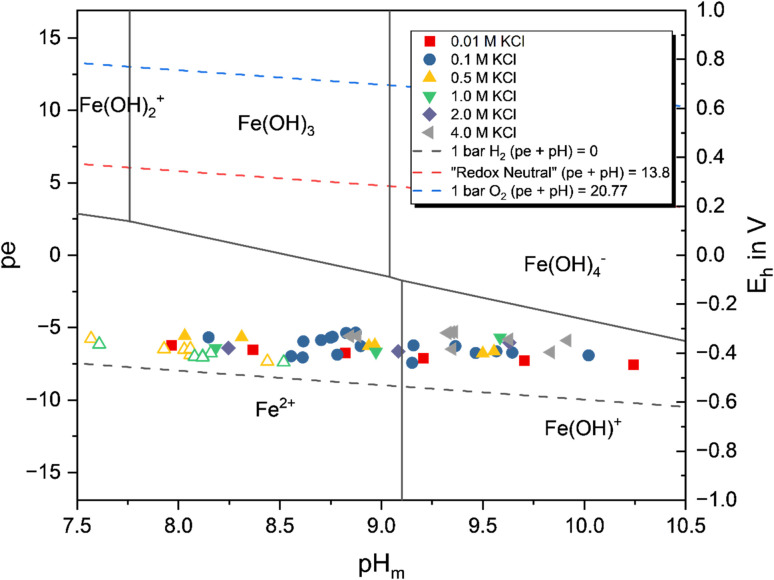
Pourbaix diagram of aqueous Fe speciation calculated in 0.1 M KCl using the data summarized in Table 1. Symbols represent the experimentally measured *E*_h_ and pH_m_ values in all investigated systems. Filled symbols represent Fe(OH)_2_(cr) systems, hollow symbols represent Fe_2_(OH)_3_Cl(cr) systems.

Fig. 3 shows the (pe + pH) measurement plotted in the Pourbaix diagram of Fe calculated using the data listed in Table 1. The data collected show that all samples are located below the borderline of the Fe(ii)/Fe(iii) redox couple (where [Fe(ii)]/[Fe(iii)] = 1), thus hinting at the predominance of Fe(ii) in solution. This behavior is further supported by the results obtained from CE-ICP-MS for selected samples, summarized in Table SI 5, which confirms the predominance (89–98%) of Fe(ii) in the aqueous phase.

**Fig. 4:**
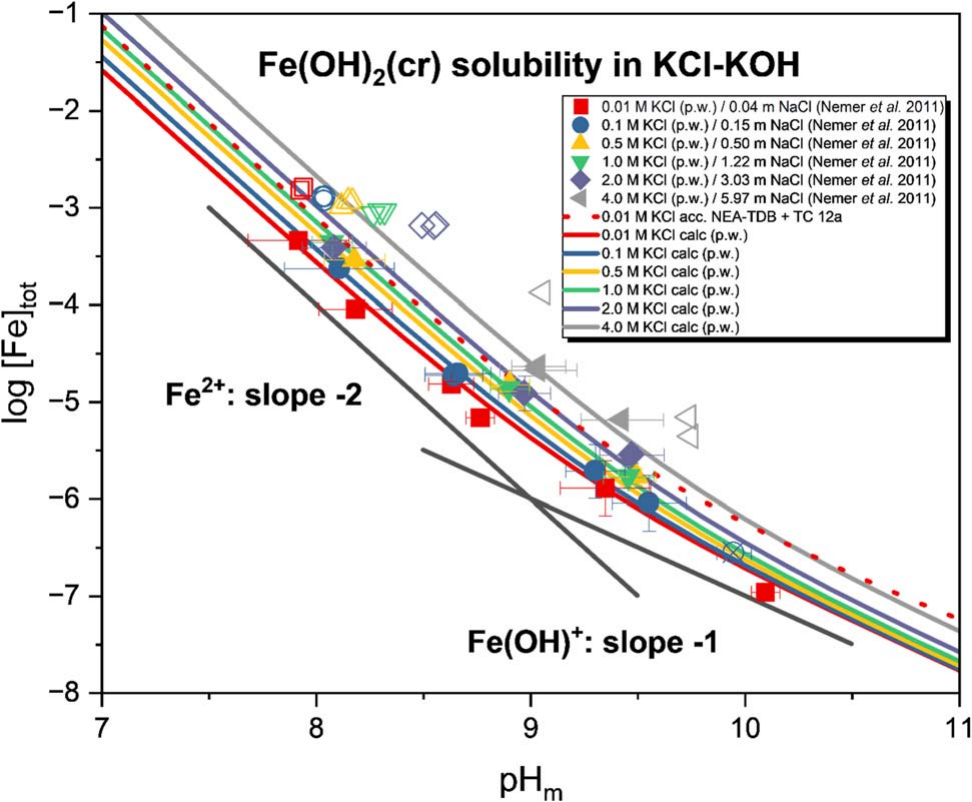
Experimental solubility of Fe(OH)_2_(cr) in 0.01–4.0 M KCl solution. Solid datapoints represent a single sample. The uncertainty of the data was calculated based on the average of different samplings (pH_m_, [Fe] measurements) with one standard deviation. The crossed data point represents [Fe] from one measurement only. Solid lines show the calculated solubilities based on the model derived in this study. The red dotted solubility curve was calculated for 0.01 M KCl using selected reference data (Table 1). Hollow symbols show solubility data collected by Nemer *et al.*^6^ in 0.04–5.97 m NaCl solutions, filled symbols were used for the data obtained within the present work.

Fig. 4 shows the experimental solubility data obtained for Fe(OH)_2_(cr) in 0.01–4.0 M KCl/KOH solutions with 7.6 ≤ pH_m_ ≤ 10.2. The data obtained follows a decreasing trend in all investigated systems with increasing pH_m_, subsequently following a slope of −2 and −1 for log[Fe]_tot_*vs.* pH_m_. This slope analysis indicates the involvement of two and one H^+^, respectively, in the equilibrium reactions controlling the Fe(ii) solubility under the investigated conditions. Considering a solubility control by Fe(OH)_2_(cr), the slope analysis of the solubility data is consistent with the equilibrium reactions (4) and (5):

4 Fe(OH)_2_(cr) + 2H^+^ ⇌ Fe^2+^ + 2H_2_O(l)

5 Fe(OH)_2_(cr) + H^+^ ⇌ FeOH^+^ + H_2_O(l)

At pH_m_ < 9, where mainly Fe^2+^ is present, the solubility follows a slope close to −2, while a flattening of this slope can be observed at pH_m_ > 9 with increasing contribution of the first hydrolysis species, *i.e.*, FeOH^+^. Note that iron concentrations above pH_m_ ≈ 9.5 could only be determined for systems with ionic strength below 0.1 M KCl, due to the higher effective detection limit at higher salt concentrations (which require a greater dilution factor). Experimental solubility data determined in this work are clearly lower (∼0.6 log-units) than the solubility calculated with reference thermodynamic data summarized in Table 1 (dotted red line in Fig. 4, corresponding to the solubility in *I* = 0.01 M KCl). Such discrepancy is expectedly due to differences in particle size of the solid phases used in the solubility experiments. An even larger deviation is observed when comparing our results with solubility data collected by Nemer *et al.*^12^ in 0.04–5.97 m NaCl solutions (empty symbols in Fig. 4). Rietveld analysis of the XRD data kindly provided by the authors revealed the less crystalline character of the solid phase used in their study (see Table SI 4). These observations highlight the importance of an accurate solid phase characterization when deriving the thermodynamic properties of solid phases in aqueous systems, in particular with regard to the relevant contribution of the surface energy to the overall 
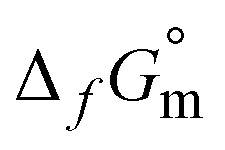
 for solid phases with particle size in the nm scale (see discussion in Neck *et al.*^36^).

#### Fe_2_(OH)_3_Cl(cr)

**Fig. 5:**
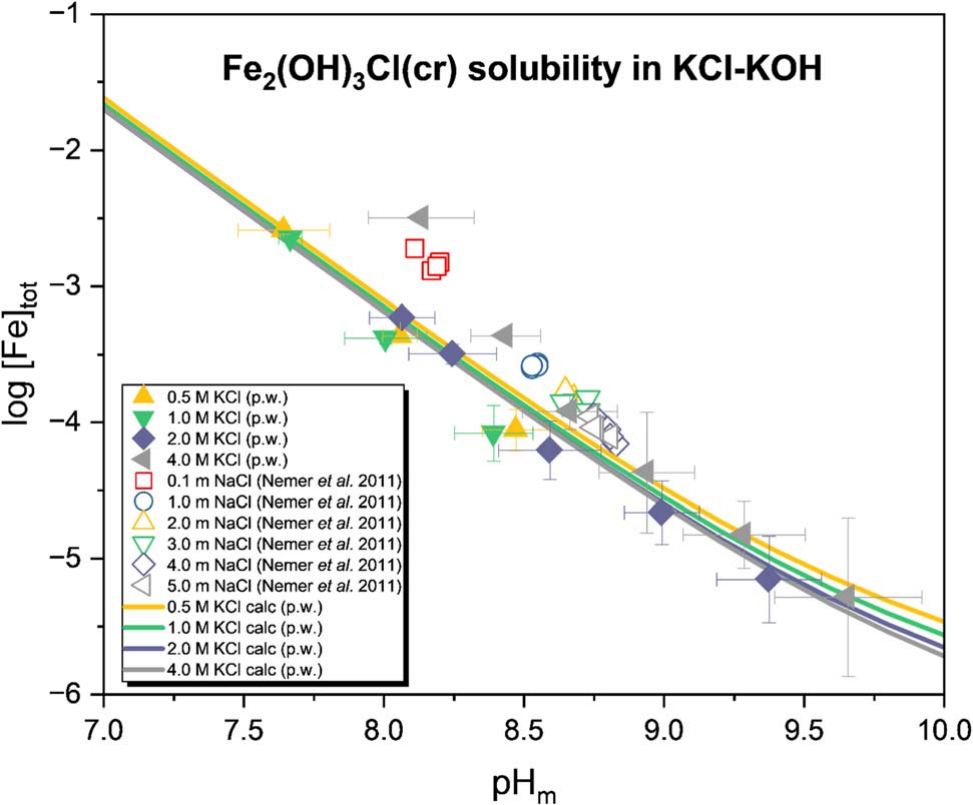
Experimental solubility of Fe_2_(OH)_3_Cl(cr) in 0.5–4.0 M KCl solution. Solid datapoints represent a single sample. The uncertainty of the data was calculated based on the average of different samplings (pH_m_, [Fe] measurements) with one standard deviation. Solubility curves were calculated based on the model derived in this study. Hollow symbols show solubility data collected by Nemer *et al.*^6^ in 0.1–5.0 m NaCl solutions, filled symbols were used for the data obtained within the present work.

Fig. 5 displays the aqueous Fe concentration determined for the samples (*I* = 0.5–4.0 M KCl) prepared with the initial solid phase Fe_2_(OH)_3_Cl(cr). (pe + pH) (Fig. 3) and CE-ICP-MS measurements (Table SI 5) confirm the predominance of Fe(ii) (96–100%) in the aqueous phase. As the stability field of Fe_2_(OH)_3_Cl(cr) shrinks with decreasing chloride concentration, samples prepared with *I* < 4.0 M, specifically at pH_m_ > 8.5 (for 0.5 and 1.0 M KCl) or pH_m_ > 9.5 (for 2.0 M KCl), exhibited (partial) transformation to Fe(OH)_2_(cr). Since it is uncertain whether the solubility in these samples is controlled by Fe_2_(OH)_3_Cl(cr) or Fe(OH)_2_(cr) (or both), the data obtained under the abovementioned conditions were excluded from the thermodynamic modelling.

The dissolution of Fe_2_(OH)_3_Cl(cr) under near-neutral to weakly alkaline pH conditions can be described according to eqn (6). A decreasing solubility with increasing pH was observed, independent of the ionic strength. At pH_m_ ≈ 7.5–8.5 the solubility was observed to be virtually independent of the ionic strength up to 2 M KCl. The log[Fe]_tot_ decreased from around −2.6 at pH_m_ ≈ 7.6 to −4.2 at pH_m_ ≈ 8.6. However, in the samples at *I* = 4.0 M KCl, the solubility was observed to be up to 0.9 log units higher at pH_m_ ≈ 8.0 compared to the systems with lower ionic strengths. This difference decreases at higher pH to around 0.3 log units between the observed solubility in the 2.0 M and 4.0 M KCl systems at pH_m_ ≈ 9.0–9.5.

6 Fe_2_(OH)_3_Cl(cr) + 3H^+^ ⇌ 2Fe^2+^ + Cl^−^ + 3H_2_O(l)

Compared to the solubility data reported by Nemer *et al.*^12^ and later selected in the NEA-TDB,^3^ an up to 0.5 log units lower solubility was observed in this study. Hagemann *et al.*^37^ investigated the solubility of Fe-Hibbingite at 25–40 °C and 6.21 ≤ −log cH ≤ 7.27, where cH corresponds to the H^+^ concentration in molar units. Three samples were prepared from oversaturation conditions by mixing aqueous solutions of FeCl_2_ and NaOH to obtain the targeted Fe-Hibbingite. An additional sample was prepared by adding iron powder to 2 m FeCl_2_ solution, which resulted in the formation of Fe-Hibbingite. Significantly higher Fe(ii) concentrations were reported by the authors (0.06–2.0 m), mainly due to the lower pH-regime considered in those experiments. The interpretation of this data set requires the consideration of Fe^2+^ as a major cation with the consequent ion–ion interactions, which are beyond the scope of this work.

### Model development from solubility experiment data

Data collected within the undersaturation solubility experiments with well-characterized Fe(OH)_2_(cr) and Fe_2_(OH)_3_Cl(cr) solid phases were used to derive solubility constants at *I* = 0 for the dissolution of those solids according to eqn (4) and (6). The conditional 
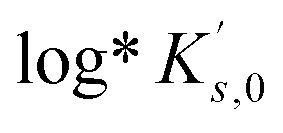
 were defined as follows:7
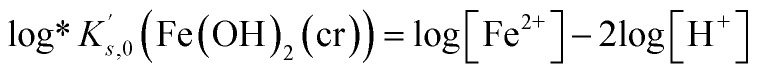
8
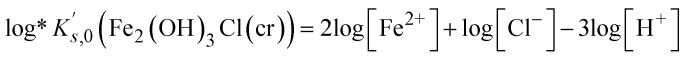


Solubility constants in the reference state 
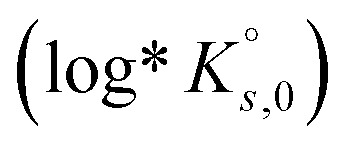
 can be calculated as described in eqn (9) and (10), where the activity coefficients *γ*_*i*_ are calculated by SIT as described above:9
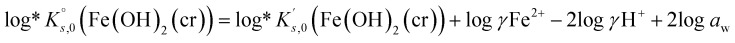
10
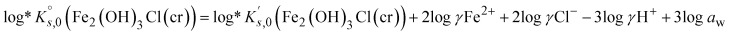


Solubility constants were derived including Fe^2+^, FeOH^+^ and FeCl^+^ as iron(ii) species prevailing in the aqueous phase. The squared residual (*R*^2^) between the measured ([Fe]_exp_) and calculated ([Fe]_calc_ = [Fe^2+^]_calc_ + [FeOH^+^]_calc_ + [FeCl^+^]_calc_) total iron concentrations was calculated for each sample. The sum of these squared residuals (SSR) ∑(log [Fe]_exp_−log [Fe]_calc_)^2^ was minimized by varying 
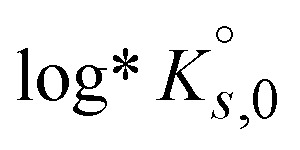
 whilst fixing the complexation constants and SIT coefficients as reported in Tables 1 and SI 7, respectively. Although the Fe(ii) chloro complex was included in the model, this has a nearly negligible contribution to the overall solubility, *i.e.*, < 4% in the 4.0 M KCl solutions investigated in this work. This observation is in line with the discussion provided in the NEA-TDB.^2^ Solubility constants in the reference state determined as described above resulted in 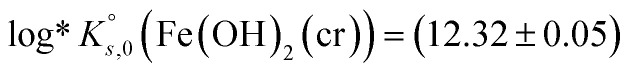 and 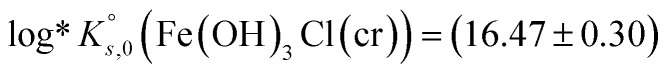, where uncertainties were calculated for 95% confidence interval using F statistics for one parameter and 17 (Fe(OH)_2_(cr)) or 24 (Fe_2_(OH)_3_Cl(cr)) datapoints.

Experimental results obtained for the systems containing both solid phases can be used for an independent validation of the thermodynamic constants derived above. The chemical equilibrium between both solid phases is defined as:

11 Fe_2_(OH)_3_Cl(cr) + H_2_O ⇌ 2Fe(OH)_2_(cr) + H^+^ + Cl^−^

Based on reaction (11), the correlation between 
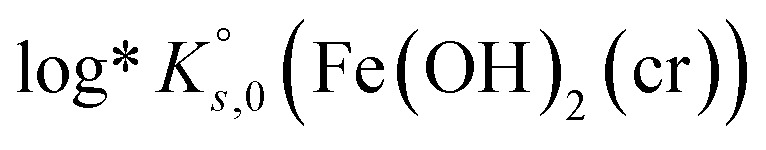
 and 
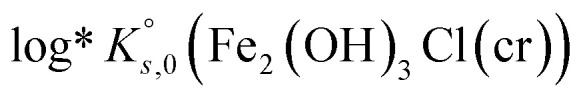
 is defined independently of Fe(ii) aqueous concentration and as a function of [H^+^] and [Cl^−^]:12
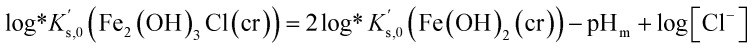


**Fig. 6:**
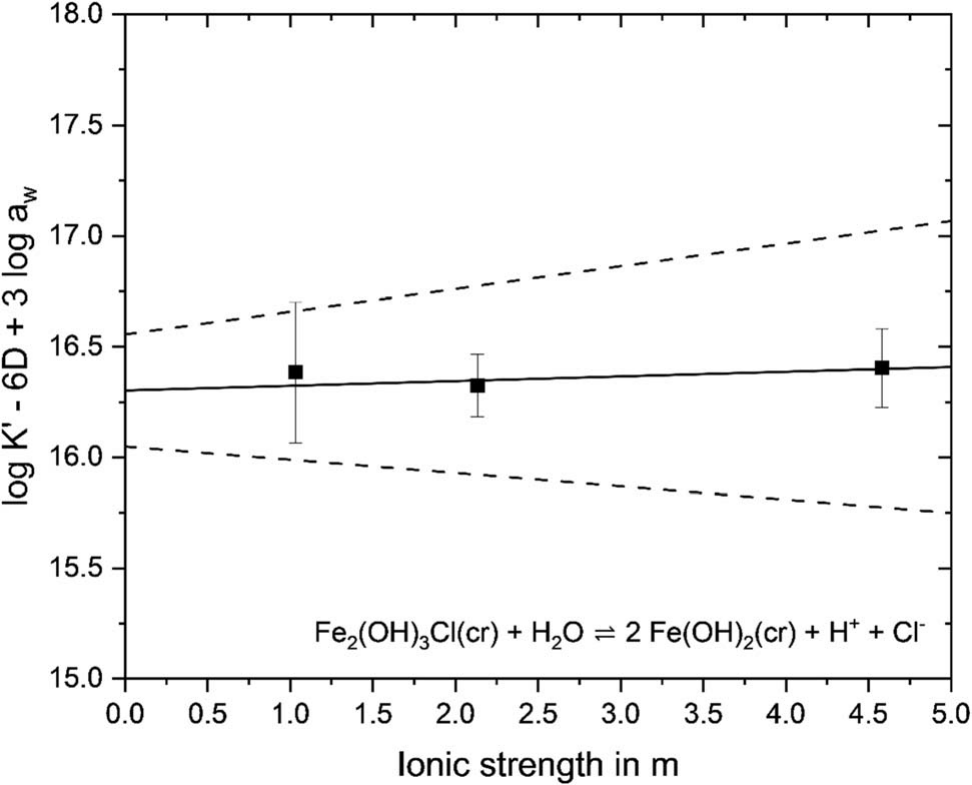
SIT plot to derive 
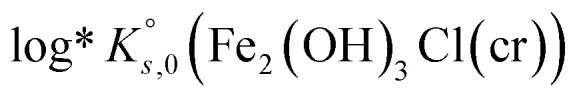
 from mixed system data where both solid phases were still present after equilibration given with two standard deviations of the averaged data from three samplings as uncertainty. Solid line represents weighted linear regression calculated according to NEA-TDB guidelines,^2^ dashed line represents the uncertainty range based on the uncertainty calculated for *I* = 0 m. Resulting in 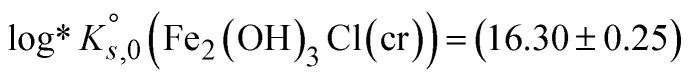 and −Δ*ε* = 0.02 ± 0.08 (*ε*(Fe^2+^, Cl^−^) = 0.17).

Using 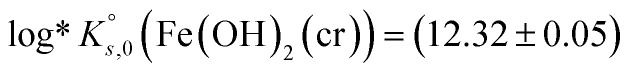 derived from the solubility experiments in the absence of Fe_2_(OH)_3_Cl(cr) (see above), and considering pH_m_ measurements and salt concentrations in the mixed systems (see Table SI 6) where both phases were still present after equilibration (*i.e.*, *I* = 1.0, 2.0, 4.0 M KCl), conditional solubility constants 
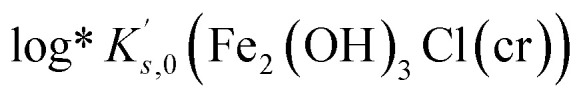
 were derived for each ionic strength, as reported in Table SI 8. Extrapolation to *I* = 0 was achieved with the SIT-plot (see Fig. 6), resulting in 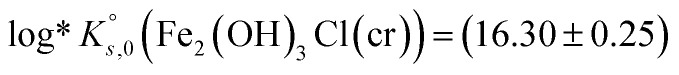, which is in excellent agreement with the solubility product determined from the experiments conducted with a single solid phase.

This validation using an independent dataset provides additional confidence in the thermodynamic constants derived in this work. The final value selected as the solubility product of hibbingite is the weighted average^2^ of the individual values obtained from solubility experiments using Fe_2_(OH)_3_Cl(cr) and a combination of Fe_2_(OH)_3_Cl(cr) + Fe(OH)_2_(cr).
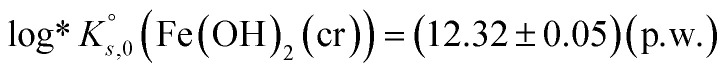

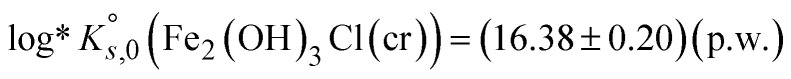


The solubility product 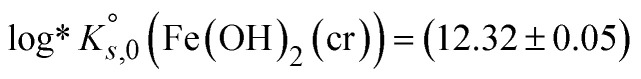 determined in this work lies well in the uncertainty range of the date selected in the ThermoChimie database^10^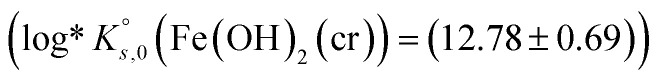 and is in excellent agreement to the data selected in the PSI/Nagra Chemical Thermodynamic Database^11^ for this solid phase 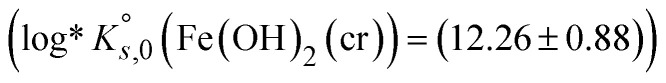. Compared to the solubility constant derived by Nemer *et al.*^6^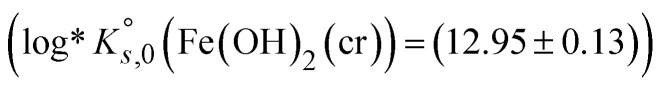, a significantly lower solubility was observed, resulting in a deviation of around 0.6 log units. For 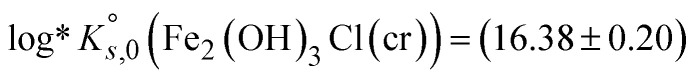, an even higher deviation was observed (*ca.* 0.8 log units). As discussed above for Fe(OH)_2_(cr), this is expectedly due to the use of solid phases with larger particle size in the present study, thus highlighting the role of the Ostwald ripening and its impact on the thermodynamic properties of solid phases forming in aqueous systems. The values for 
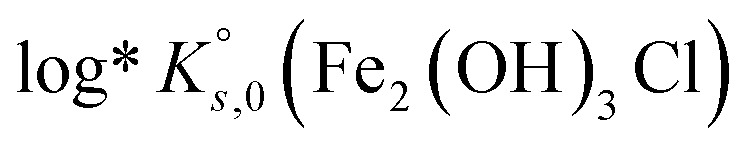
 reported by Hagemann *et al.*^37^ at the end of their experiments at 25 °C range from 17.17 (oversaturation experiments) to 18.09 (undersaturation experiments with Fe(0) powder), which are accordingly *ca.* 0.8 and 1.7 log units higher than the values determined in this study. Although no XRD data were provided in the report, oversaturation experiments may have resulted in less crystalline solid phases, possibly explaining the differences in 
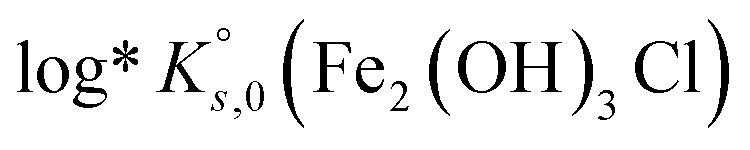
.

### Environmental implications

The thermodynamic models derived in this work for Fe(OH)_2_(cr) and Fe_2_(OH)_3_Cl(cr) can be used as a basis to assess the (geo-)chemical boundary conditions in which these solid phases are to be expected in the context of underground repositories for nuclear waste disposal. Investigations in pure KCl solutions allow isolating and understanding the fundamental thermodynamic and speciation behavior of iron. In this respect, Pourbaix diagrams arise as a helpful tool to identify those solid phases expected to form through the anoxic corrosion of iron components, and thus responsible for buffering the redox conditions in the repository, *e.g.*, Fe(cr)/Fe(OH)_2_(cr), Fe(OH)_2_(cr)/Fe_3_O_4_(cr), among others. However, this exercise requires an appropriate knowledge of these solid phases and their thermodynamic properties. A common but less acknowledged pitfall is the combined use of thermodynamic constants involving solid phases that greatly differ in their crystallinity degree and/or particle size. This approach tends to overrepresent the more crystalline solid phases, potentially inducing great deviations in the (pe + pH) borderlines defined by a given redox couple. Solid phases with well-ordered crystalline structures and large particle size are often synthesized at very high temperatures. They are most appropriate to derive true (bulk) thermodynamic properties of these materials through thermochemical studies (*e.g.*, calorimetry), but their formation is often kinetically hindered in aqueous systems under ambient conditions, and they neglect the contribution of the surface energy to the overall 
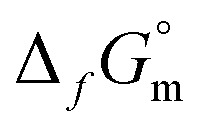
 of the solid. This is particularly relevant for strongly hydrolyzing metal ions like M(iii) and M(iv), for which amorphous or nanocrystalline solid phases might be responsible for the solubility control in aqueous systems. Note, however, that in the thermal phase of a repository lasting for thousands of years, elevated temperatures exist, which may easily lead to the formation of solid phases with a higher degree of crystallinity.

**Fig. 7:**
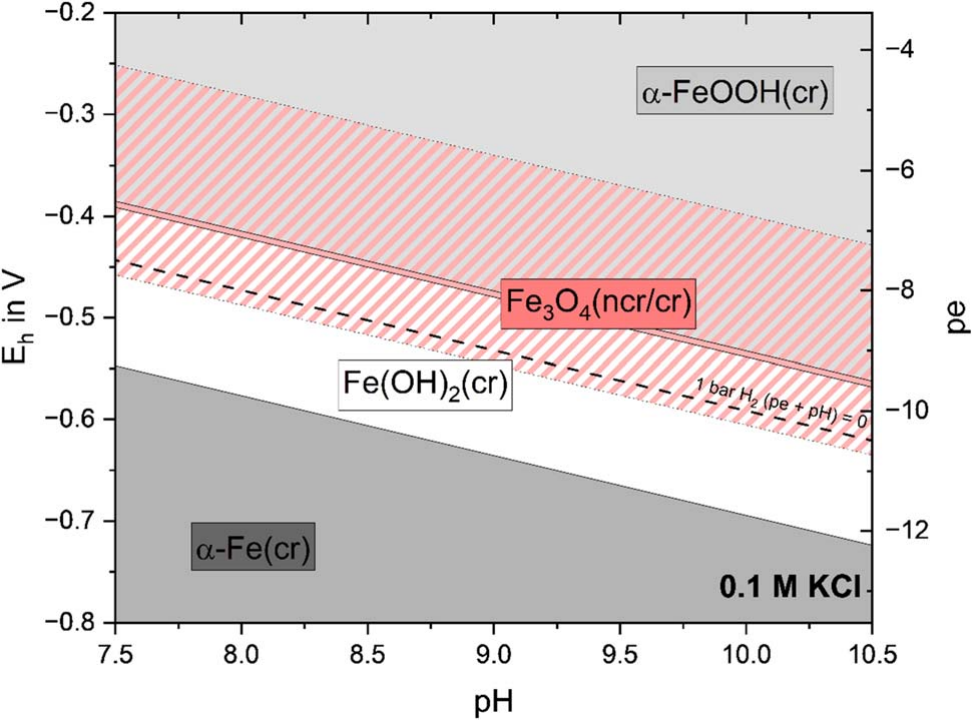
Pourbaix diagram (calculated with activities) for solid phases in 0.1 M KCl using solubility constants selected in the ThermoChimie database^10^ (α-Fe(cr), Fe_3_O_4_(cr) (red-striped area), α-FeOOH(cr)), reported by Bruno *et al.*^16^ (Fe_3_O_4_(ncr) (solid red area)), and generated in this work (Fe(OH)_2_(cr)).

Fig. 7 illustrates the Pourbaix diagram of Fe calculated within 7.5 ≤ pH ≤ 10.5 and −13.5 ≤ pe ≤ −3.5 in 0.1 M KCl. Four different solid phases are expected under these conditions: α-Fe(cr), Fe(OH)_2_(cr), Fe_3_O_4_(ncr/cr) and α-FeOOH(cr). The red-striped and solid red areas represent the predominance fields of crystalline Fe_3_O_4_(cr) and nanocrystalline Fe_3_O_4_(ncr), respectively. Thermodynamic data used for the calculation are based on thermochemical studies (α-Fe(cr), Fe_3_O_4_(cr), α-FeOOH(cr))^10^ as well as solubility studies with solid phases synthesized under ambient conditions as done in this study (Fe(OH)_2_(cr) (p.w.), Fe_3_O_4_(ncr)^16^). When crystalline Fe_3_O_4_(cr) is included in the calculation, the stability field of Fe(OH)_2_(cr) is narrow and prevails exclusively beyond the stability field of water (dashed black line). According to the diagram, the couple Fe(OH)_2_(cr)/Fe_3_O_4_(cr) buffers the redox conditions at (pe + pH) ≈ −0.5. A significantly greater stability field of Fe(OH)_2_(cr) is expected at the expense of Fe_3_O_4_ stability when the nanocrystalline Fe_3_O_4_(ncr) is used in the calculation. In this case, the redox buffering defined by the couple Fe(OH)_2_(cr)/Fe_3_O_4_(ncr) results in significantly less reducing conditions, *i.e.*, (pe + pH) ≈ +1. However, note that no significant transformation from Fe(OH)_2_(cr) to Fe_3_O_4_(cr) phases could be observed under the ambient conditions in the absence of oxygen within this study.

Beyond the implications with regard to the corrosion products to be expected under repository conditions, this discussion has direct consequences for the prediction of the chemical behavior of redox-sensitive radionuclides (*e.g.*, Pu, Tc, Se, *etc.*) or chemotoxic elements (*e.g.*, Cr, Pb). In connection with the Pourbaix diagrams discussed above, the predominance of only Pu(iii) is to be expected at (pe + pH) ≈ −0.5, whereas the predominance of both Pu(iii) and Pu(iv) is foreseen at (pe + pH) ≈ +1 (depending upon pH).^38–40^ A modified version of Fig. 7 including the redox borderline in the aqueous system between Pu(iii) and Pu(iv) is shown in Fig. SI 3. Not only consistent thermodynamic data, but also a consistent set of solid phases, as well as proper knowledge about relevant redox reactions, is required to make reliable predictions in the context of nuclear waste disposal and beyond, for systems of environmental relevance.

## Conclusion

Undersaturation solubility experiments with two well-defined Fe(ii) solid phases (*i.e.*, Fe(OH)_2_(cr) and Fe_2_(OH)_3_Cl(cr)) in near-neutral to alkaline pH conditions were performed in dilute to concentrated KCl solutions. The experimental data were used to develop chemical, thermodynamic and activity models that enable reliable predictions of the stability of iron corrosion phases relevant to underground nuclear waste repositories. Extensive phase characterization by XRD combined with Rietveld analysis provided detailed insights into their structural composition and ensured an accurate description of the solubility-controlling phases and their long-term stability. In this way, it directly addresses the criticism discussed in NEA-TDB regarding the insufficient phase characterization in previous studies. The demonstrated stability of Fe(ii) solid phases has important implications for post-closure redox buffering, as it constrains the Fe(ii)/Fe(iii) stability boundary and thereby influences the chemical behavior of redox-sensitive radionuclides and chemotoxic elements.

## Author contributions

P. Q. Fürst: writing – review & editing, writing – original draft, methodology, investigation, formal analysis. N. Çevirim-Papaioannou: writing – review & editing, supervision, methodology. X. Gaona: writing – review & editing, supervision, project administration, funding acquisition, conceptualization. K. Garbev: writing – review & editing, methodology, investigation, formal analysis. T. Roth: writing – review & editing, methodology, investigation. S. Hagemann: writing – review & editing, investigation, methodology. M. Altmaier: writing – review & editing, project administration, funding acquisition, conceptualization. H. Geckeis: writing – review & editing, supervision.

## Conflicts of interest

There are no conflicts to declare.

## Note added after first publication

This article replaces the version published on 25 Nov 2025, which contained errors in Table 2.

## Supplementary Material

RA-015-D5RA07073B-s001

## Data Availability

The data supporting this article have been included as part of the supplementary information (SI). Ref. 41 is cited in the SI. Supplementary information is available. See DOI: https://doi.org/10.1039/d5ra07073b.

## References

[cit1] Ziemniak S. E., Jones M. E., Combs K. E. S. (1995). Magnetite Solubility and Phase Stability in Alkaline Media at Elevated Temperatures. J. Solution Chem..

[cit2] NEA , Chemical Thermodynamics of Iron, Part 1, OECD Publishing, Paris, 2013

[cit3] NEA , Chemical Thermodynamics of Iron, Part 2, OECD Publishing, Paris, 2020

[cit4] Leussing D. L., Kolthoff I. M. (1953). The Solubility Product of Ferrous Hydroxide and the Ionization of the Aquo-Ferrous Ion. J. Am. Chem. Soc..

[cit5] Refait P., Bon C., Simon L., Bourrié G., Trolard F., Bessière J., Gènin J.-M. R. (1999). Chemical Composition and Gibbs Standard Free Energy of Formation of Fe(II)-Fe(III) Hydroxysulphate Green Rust and Fe(II) Hydroxide. Clay Miner..

[cit6] Nemer M. B., Xiong Y., Ismail A. E., Jang J.-H. (2011). Solubility of Fe2(OH)3Cl (Pure-Iron End-Member of Hibbingite) in NaCl and Na2SO4 Brines. Chem. Geol..

[cit7] Dauphin J., Dauphin S., Chatonier D., Vialatte M.-T. (1964). L’hydroxyde ferreux (3e note). Produit de solubilité relatif à l’ion Fe++ et complexes ferro-chlorés. Bull. Soc. Chim. Fr..

[cit8] Chase M. W. , NIST-JANAF Thermochemical Tables, American Institute of Physics, 1998

[cit9] Chivot J. , Thermodynamique des produits de corrosion: fonctions thermodynamiques, diagrammes de solubilité, diagrammes E-ph des systèmes Fe-H2O, Fe-CO2-H2O, Fe-S-H2O, Cr-H2O et Ni-H2O en fonction de la temperature, ANDRA, 2004

[cit10] Giffaut E., Grivé M., Blanc Ph., Vieillard Ph., Colàs E., Gailhanou H., Gaboreau S., Marty N., Madé B., Duro L. (2014). Andra Thermodynamic Database for Performance Assessment: ThermoChimie. Appl. Geochem..

[cit11] Hummel W. and Thoenen T., PSI/Nagra Chemical Thermodynamic Database, Technical Report 21–03, Nagra, Wettingen, Switzerland, 2023

[cit12] Brown P. L. and Ekberg C., Hydrolysis of Metal Ions, John Wiley & Sons, 2016

[cit13] Oswald H. R., Feitknecht W. (1964). Über Die Hydroxidhalogenide Me2(OH)3Cl, -Br, -J Zweiwertiger Metalle (Me = Mg, Ni, Co, Cu, Fe, Mn). Helv. Chim. Acta.

[cit14] Koděra P., Majzlan J., Pollok K., Kiefer S., Šimko F., Scholtzová E., Luptáková J., Cawthorn G. (2022). Ferrous Hydroxychlorides Hibbingite [γ-Fe2(OH)3Cl] and Parahibbingite [β-Fe2(OH)3Cl] as a Concealed Sink of Cl and H2O in Ultrabasic and Granitic Systems. Am. Mineral..

[cit15] Kim S., Marrs C., Nemer M., Je-Hun Jang J. (2017). Solubility Model for Ferrous Iron Hydroxide, Hibbingite, Siderite, and Chukanovite in High Saline Solutions of Sodium Chloride, Sodium Sulfate, and Sodium Carbonate. ACS Earth Space Chem..

[cit16] Bruno J. , González-Siso M. R., Duro L., Gaona X. and Altmaier M., Swedish Nuclear Fuel and Waste Management Company (Svensk Kärnbränslehantering Aktiebolag, abbreviated SKB), 2018

[cit17] Rémazeilles C., Refait Ph. (2008). Formation, Fast Oxidation and Thermodynamic Data of Fe(II) Hydroxychlorides. Corros. Sci..

[cit18] Altmaier M., Metz V., Neck V., Müller R., Fanghänel Th. (2003). Solid-Liquid Equilibria of Mg(OH)2(Cr) and Mg2(OH)3Cl·4H2O(Cr) in the System Mg-Na-H-OH-Cl-H2O at 25°C. Geochim. Cosmochim. Acta.

[cit19] Baumann A., Yalçıntaş E., Gaona X., Altmaier M., Geckeis H. (2017). Solubility and Hydrolysis of Tc(iv) in Dilute to Concentrated KCl Solutions: An Extended Thermodynamic Model for Tc ^4+^ –H ^+^ –K ^+^ –Na ^+^ –Mg ^2+^ –Ca ^2+^ –OH ^−^ –Cl ^−^ –H _2_ O(l) Mixed Systems. New J. Chem..

[cit20] Pozdniakova S., Padarauskas A., Schwedt G. (1997). Simultaneous Determination of Iron(II) and Iron(III) in Water by Capillary Electrophoresis. Anal. Chim. Acta.

[cit21] Graser C.-H., Banik N. L., Bender K. A., Lagos M., Marquardt C. M., Marsac R., Montoya V., Geckeis H. (2015). Sensitive Redox Speciation of Iron, Neptunium, and Plutonium by Capillary Electrophoresis Hyphenated to Inductively Coupled Plasma Sector Field Mass Spectrometry. Anal. Chem..

[cit22] Ciavatta L. (1980). Annali di Chimica.

[cit23] Neck V., Altmaier M., Rabung T., Lützenkirchen J., Fanghänel T. (2009). Thermodynamics of Trivalent Actinides and Neodymium in NaCl, MgCl2, and CaCl2 Solutions: Solubility, Hydrolysis, and Ternary Ca-M(III)-OH Complexes. Pure Appl. Chem..

[cit24] Gaona X., Fellhauer D., Altmaier M. (2013). Thermodynamic Description of Np(VI) Solubility, Hydrolysis, and Redox Behavior in Dilute to Concentrated Alkaline NaCl Solutions. Pure Appl. Chem..

[cit25] Altmaier M., Yalçıntaş E., Gaona X., Neck V., Müller R., Schlieker M., Fanghänel T. (2017). Solubility of U(VI) in Chloride Solutions. I. The Stable Oxides/Hydroxides in NaCl Systems, Solubility Products, Hydrolysis Constants and SIT Coefficients. J. Chem. Thermodyn..

[cit26] Çevirim-Papaioannou N., Yalçıntaş E., Gaona X., Altmaier M., Geckeis H. (2018). Solubility of U(VI) in Chloride Solutions. II. The Stable Oxides/Hydroxides in Alkaline KCl Solutions: Thermodynamic Description and Relevance in Cementitious Systems. Appl. Geochem..

[cit27] Çevirim-Papaioannou N., Gaona X., Böttle M., Bethune E. Y., Schild D., Adam C., Sittel T., Altmaier M. (2020). Thermodynamic description of Be(II) solubility and hydrolysis in acidic to hyperalkaline NaCl and KCl solutions. Appl. Geochem..

[cit28] Gates-Rector S., Blanton T. (2019). The Powder Diffraction File: A Quality Materials Characterization Database. Powder Diffr..

[cit29] Gražulis S., Daškevič A., Merkys A., Chateigner D., Lutterotti L., Quirós M., Serebryanaya N. R., Moeck P., Downs R. T., Le Bail A. (2012). Crystallography Open Database (COD): An Open-Access Collection of Crystal Structures and Platform for World-Wide Collaboration. Nucleic Acids Res..

[cit30] Natta G., Casazza E. (1927). Struttura Cristallina Ed Atomica Dell’idrato Ferroso. Atti Accad. Naz. Lincei, Cl. Sci. Fis., Mat. Nat., Rend..

[cit31] Wyckoff R. W. G. , Crystal Structures, Wiley, New York, 1963

[cit32] Parise J. B., Marshall W. G., Smith R. I., Lutz H. D., Möller H. (2000). The Nuclear and Magnetic Structure of “White Rust”—Fe (OH0. 86D0. 14) 2. Am. Mineral..

[cit33] Refait P., Génin J.-M. R. (1993). The Oxidation of Ferrous Hydroxide in Chloride-Containing Aqueous Media and Pourbaix Diagrams of Green Rust One. Corros. Sci..

[cit34] Refait Ph., Abdelmoula M., GÉnin J.-M. R. (1998). Mechanisms of Formation and Structure of Green Rust One in Aqueous Corrosion of Iron in the Presence of Chloride Ions. Corros. Sci..

[cit35] Stefánsson A. (2007). Iron(III) Hydrolysis and Solubility at 25 °C. Environ. Sci. Technol..

[cit36] Neck V., Altmaier M., Seibert A., Yun J. I., Marquardt C. M., Fanghänel T. (2007). Solubility and Redox Reactions of Pu(IV) Hydrous Oxide: Evidence for the Formation of PuO 2+ *x* (s, Hyd). Radiochim. Acta.

[cit37] Hagemann S. and Mönig H., Stability of Iron Corrosion Phases Expected in a Repository in Lower Cretaceous Clay, Gesellschaft für Anlagen- und Reaktorsicherheit (GRS) gGmbH, Köln, 2021

[cit38] Tasi A., Gaona X., Fellhauer D., Böttle M., Rothe J., Dardenne K., Schild D., Grivé M., Colàs E., Bruno J., Källström K., Altmaier M., Geckeis H. (2018). Redox Behavior and Solubility of Plutonium under Alkaline, Reducing Conditions. Radiochim. Acta.

[cit39] Second Update on the Chemical Thermodynamics of U, Np, Pu, Am and Tc, Nuclear Energy Agency (NEA), https://www.oecd-nea.org/jcms/pl_46643/second-update-on-the-chemical-thermodynamics-of-u-np-pu-am-and-tc?details=true, accessed 2025-04-28

[cit40] Schramke J. A., Santillan E. F. U., Peake R. T. (2020). Plutonium Oxidation States in the Waste Isolation Pilot Plant Repository. Appl. Geochem..

[cit41] Hummel W. Ionic Strength Corrections and Estimation of SIT Ion Interaction Coefficients, Internal report TM-44-09-01, Paul Scherrer Institute (PSI), 2009

