# Supplementary material for: Solubility, speciation and thermodynamics of Fe in reducing aqueous KCl solutions
Source: RSC Adv. 2025 Nov 25;15(54):46308–19. doi: 10.1039/d5ra07073b (PMC12645375; doi:10.1039/d5ra07073b)
Supplement: RA-015-D5RA07073B-s001 [file RA-015-D5RA07073B-s001.pdf]

Supplementary Information (SI) for RSC Advances.
This journal is © The Royal Society of Chemistry 2025

Supplementary Information

Table SI 1 *The structure of $Fe(OH)_2$ is refined in the space group P-3m1 (164). The Fe^{2+} is on a special position 1a and is not refined. O^{2-} is also on a special position x= 2/3, y=1/3 but allows refinement in z. H^{+} is positioned at x=2/3, y=1/3 and z= 0.4111. Due to the limitations of XRD for H, its z position was not refined. Refinement of the z coordinate of the oxygen resulted in 0.2193(15) which is close to the 0.2213(2) as proposed by Parise et al (2000).*

Pos.	Atom	Wyckoff	*x*	*y*	*z*	Occ.	Biso
Fe1	Fe^{2+}	1 a	0	0	0	1.	1.
O1	O^{2-}	2 d	1/3	2/3	**0.2193(15)**	1.	1.
H1	H^{1+}	2 d	1/3	2/3	0.4111	1.	1.

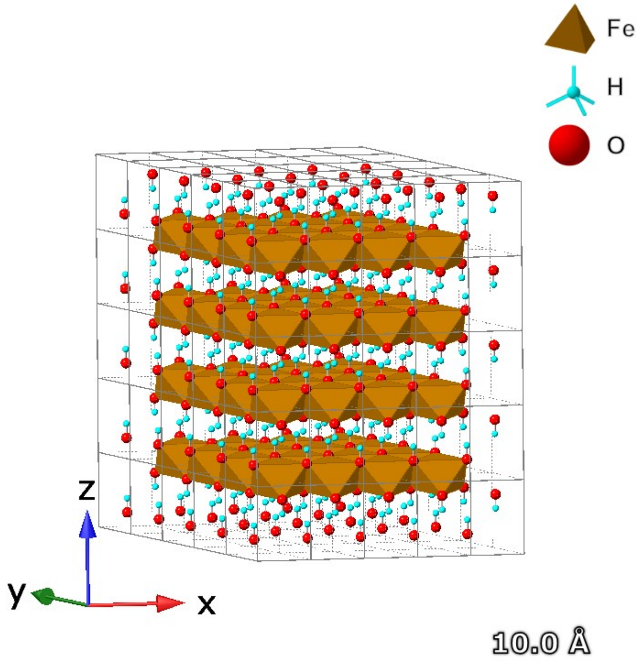

Figure SI 1. *Crystal structure of $Fe(OH)_2$.*

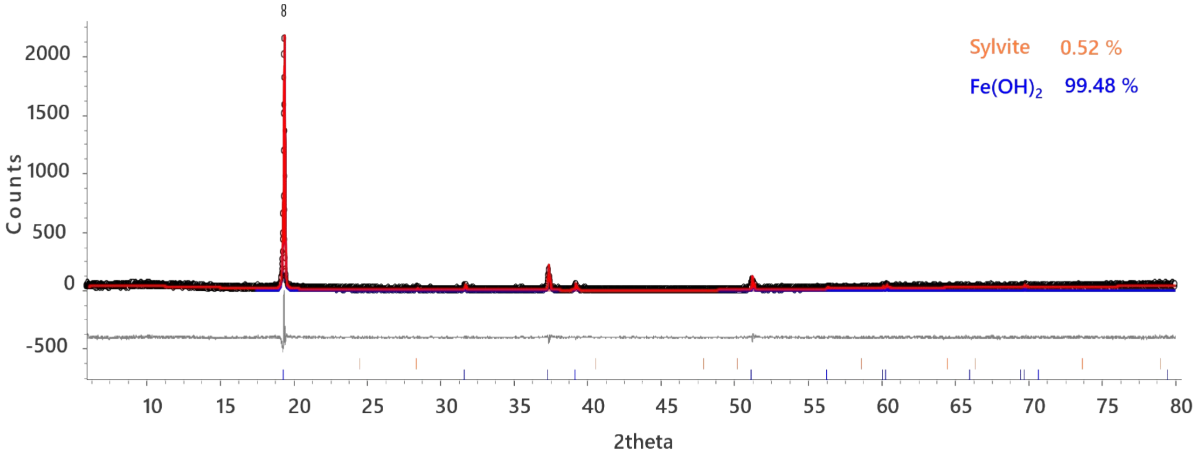

Sa

mple $Fe(OH)_2$(cr) 0.01 M KCl, pH 8

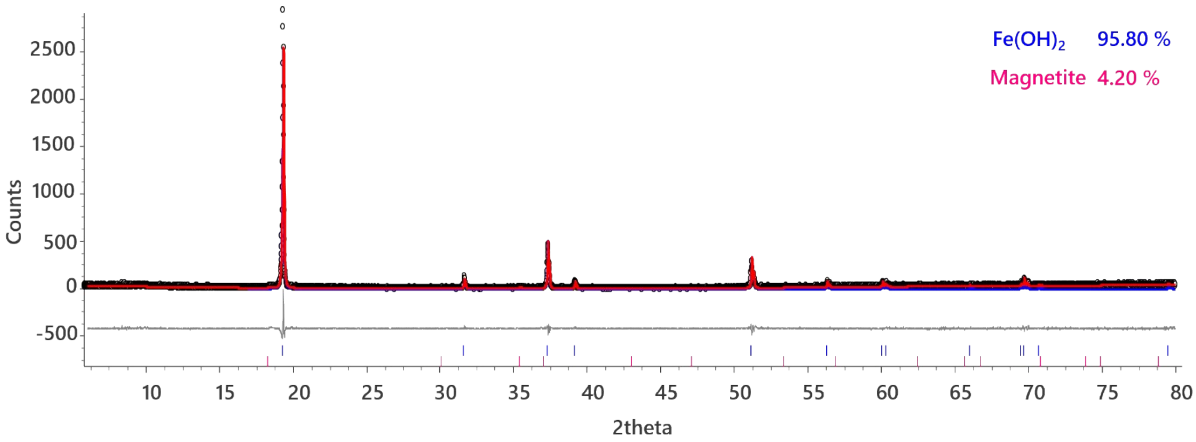

Sa

mple $Fe(OH)_2$(cr) 0.01 M KCl, pH 10

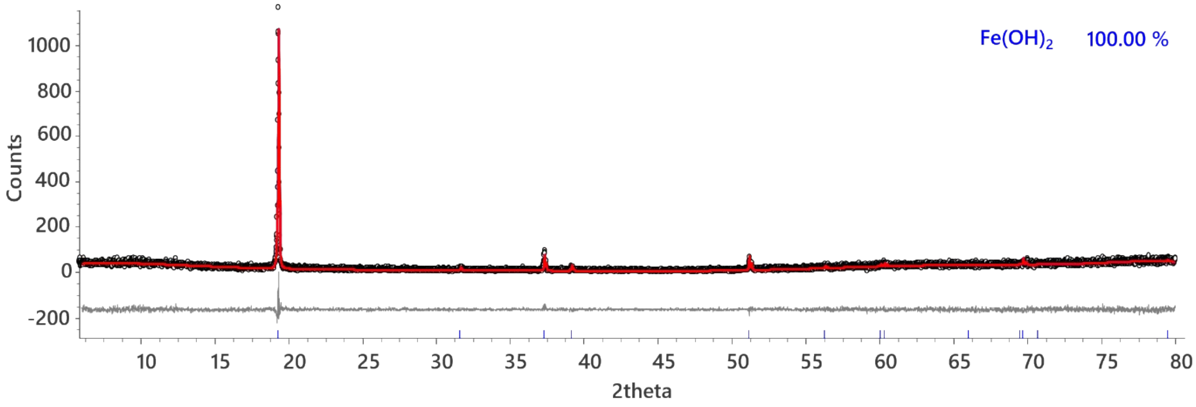

Sa

mple $Fe(OH)_2$(cr) 0.1 M KCl, pH 8

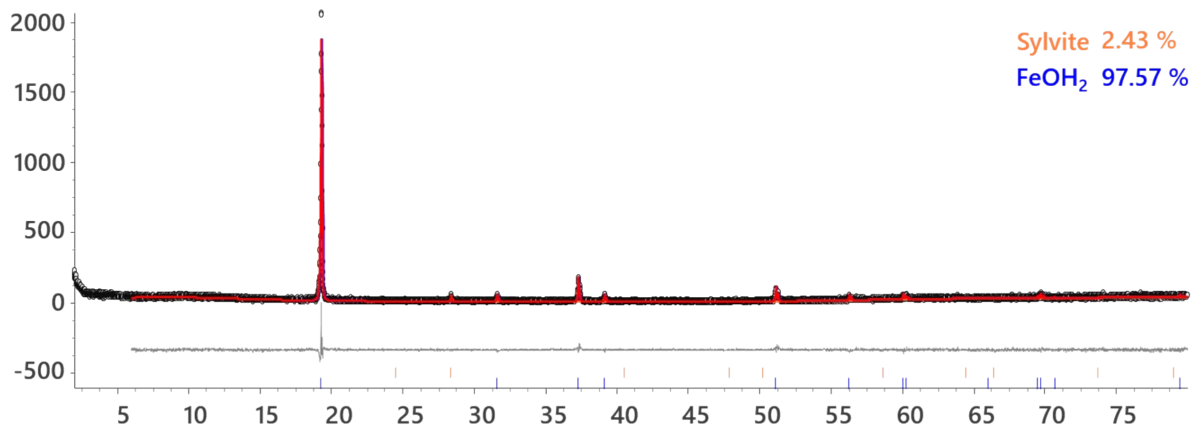

Sa

mple $Fe(OH)_2(cr)$ 0.1 M KCl, pH 10

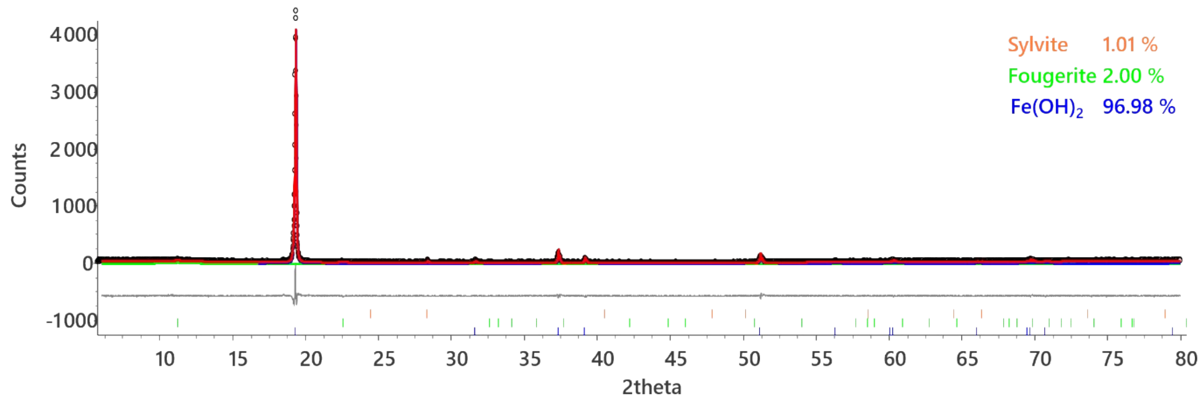

Sa

mple $Fe(OH)_2(cr)$ 0.5 M KCl, pH 8

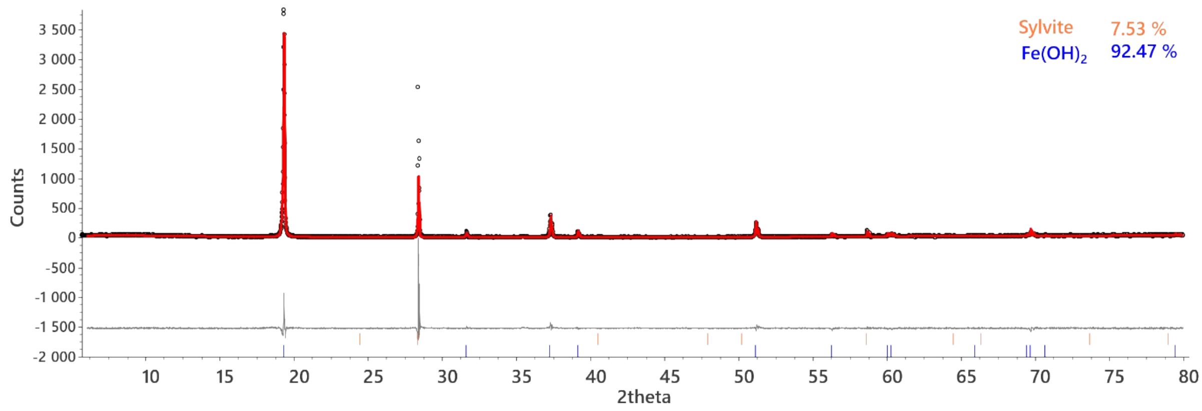

Sa

mple $Fe(OH)_2(cr)$ 0.5 M KCl, pH 10

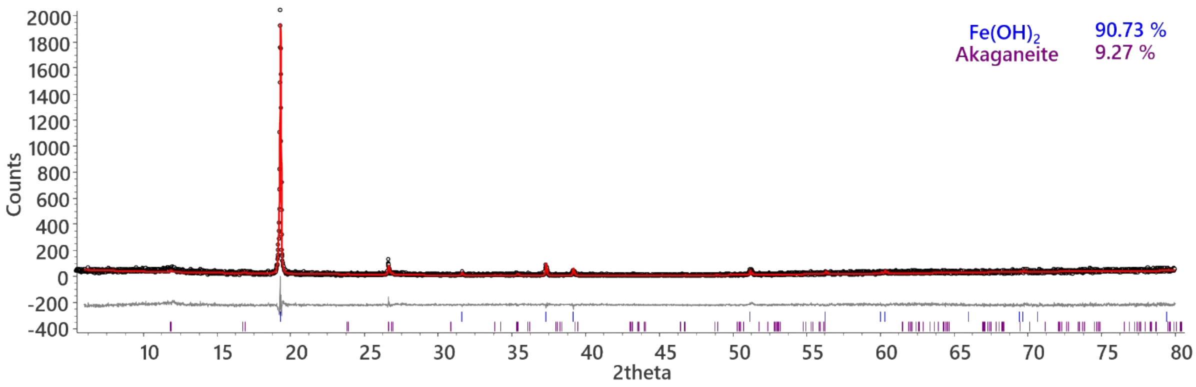

Sa

mple $Fe(OH)_2$(cr) 1.0 M KCl, pH 8

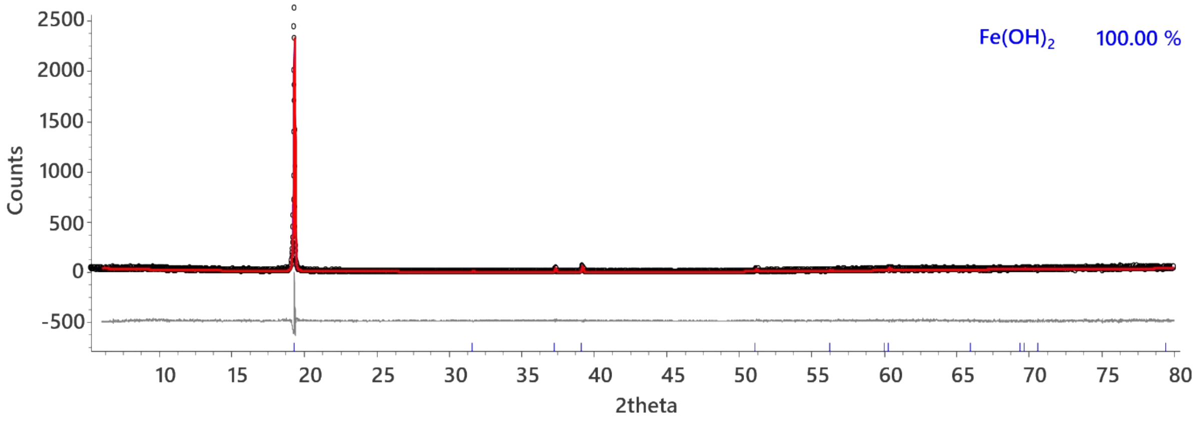

Sa

mple $Fe(OH)_2$(cr) 1.0 M KCl, pH 10

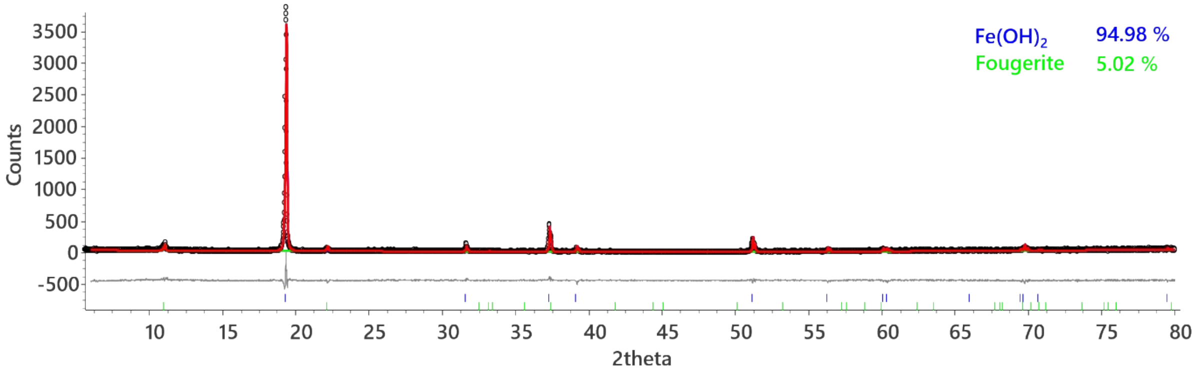

Sa

mple $Fe(OH)_2$(cr) 2.0 M KCl, pH 8

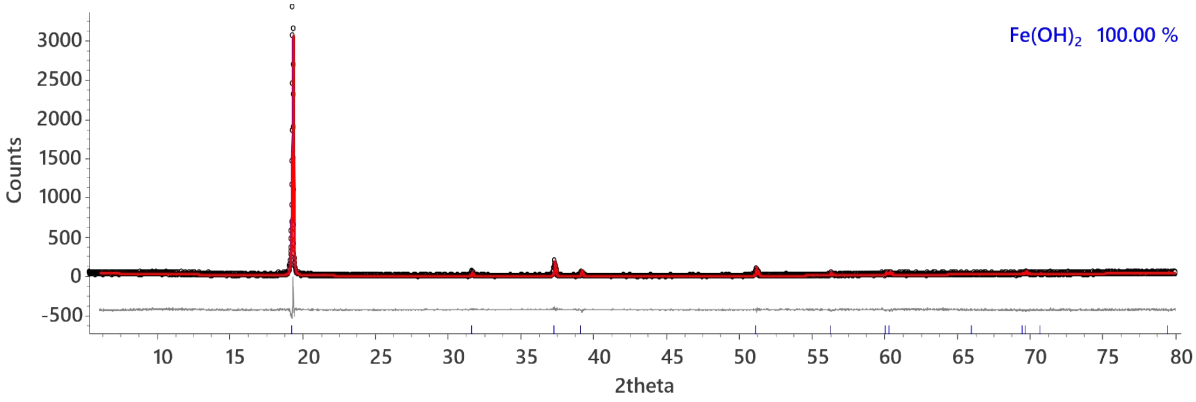

Sa

mple $Fe(OH)_2$(cr) 2.0 M KCl, pH 10

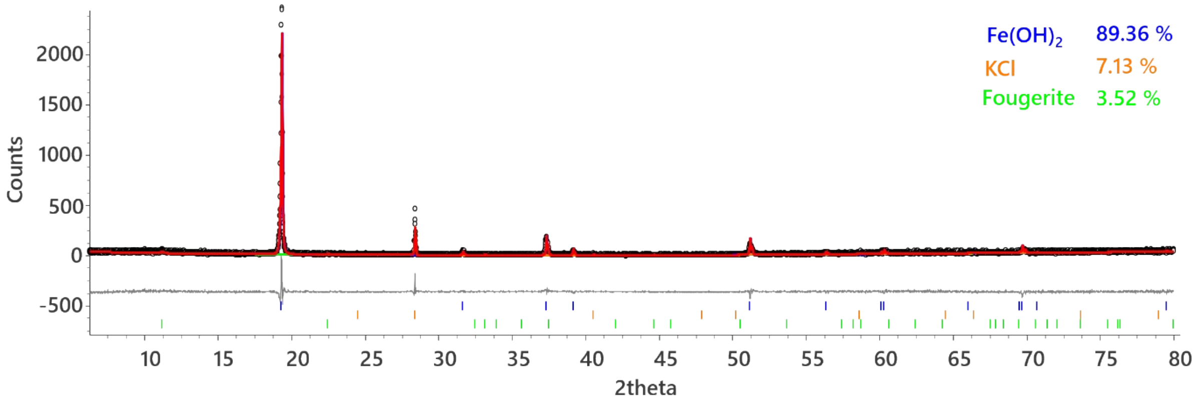

Sa

mple $Fe(OH)_2$(cr) 4.0 M KCl, pH 8

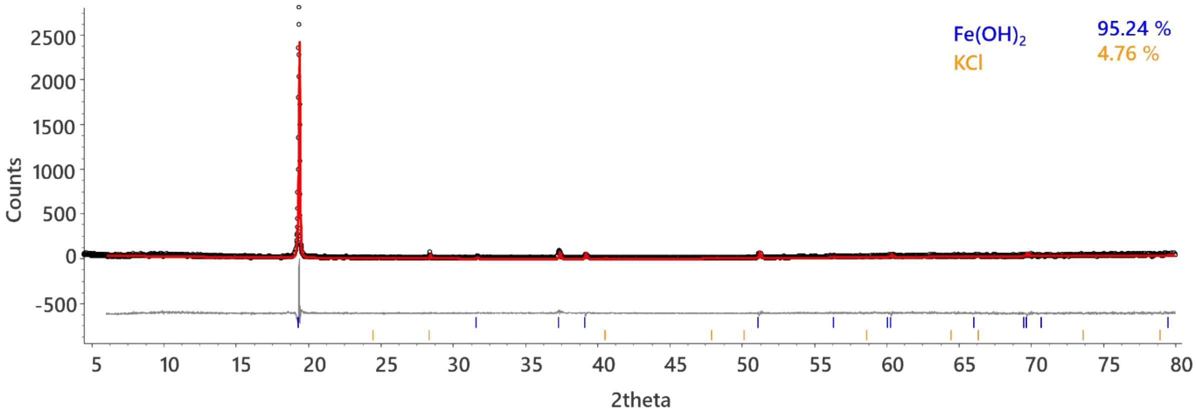

Sa

mple $Fe(OH)_2$(cr) 4.0 M KCl, pH 10

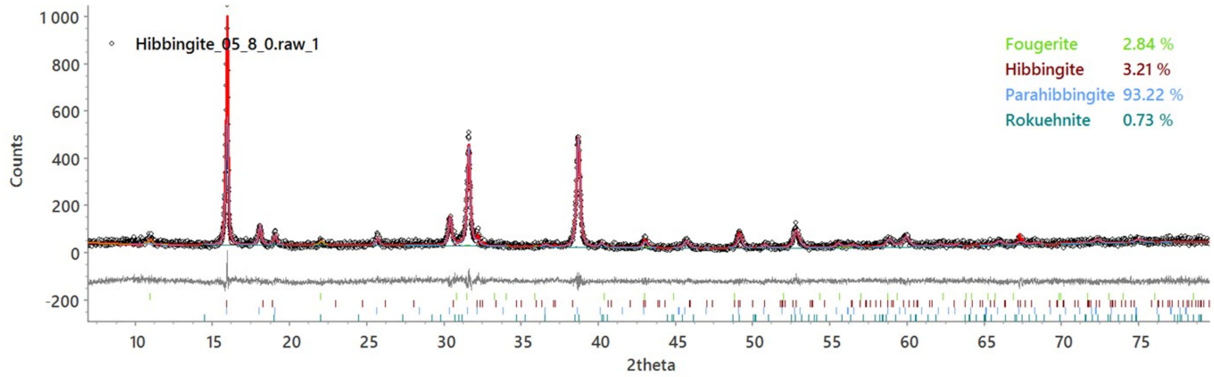

Sa

mple $Fe_2(OH)_3Cl(cr)$ 0.5 M KCl, pH 8

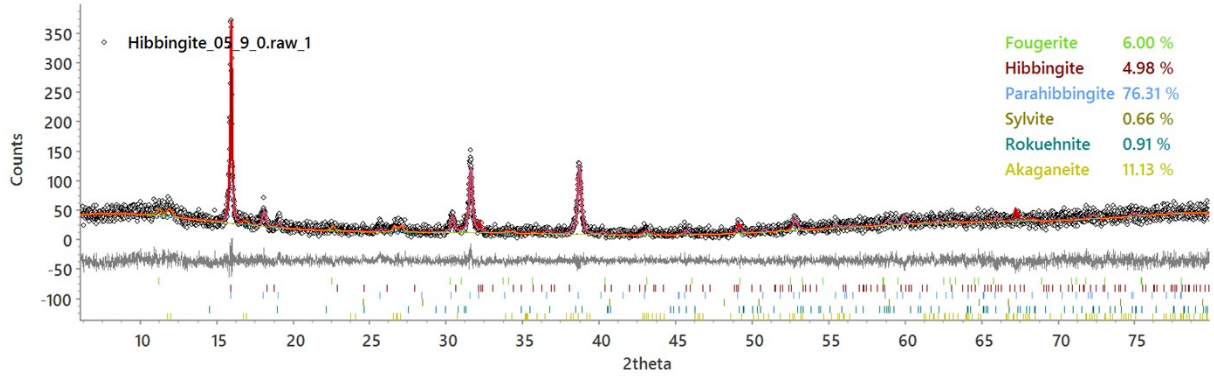

Sa

mple $Fe_2(OH)_3Cl(cr)$ 0.5 M KCl, pH 9

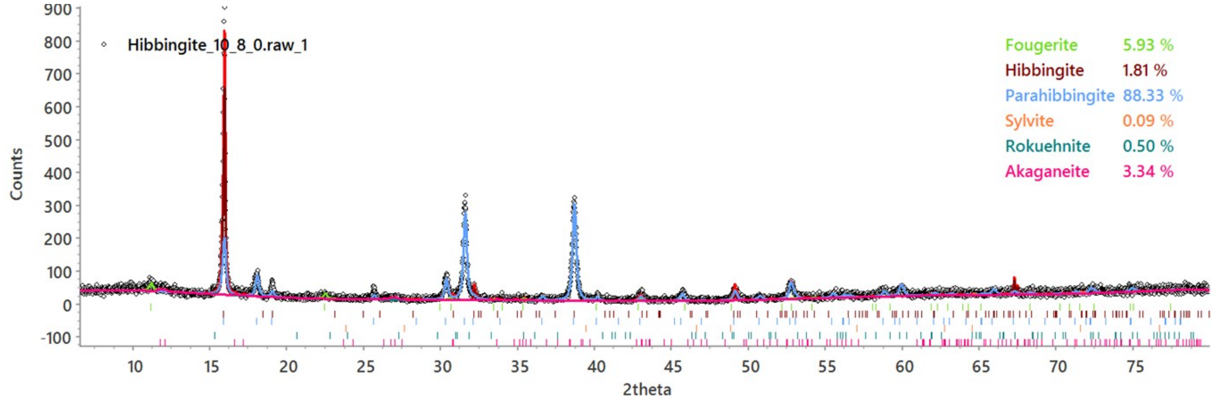

Sa

mple $Fe_2(OH)_3Cl(cr)$ 1.0 M KCl, pH 8

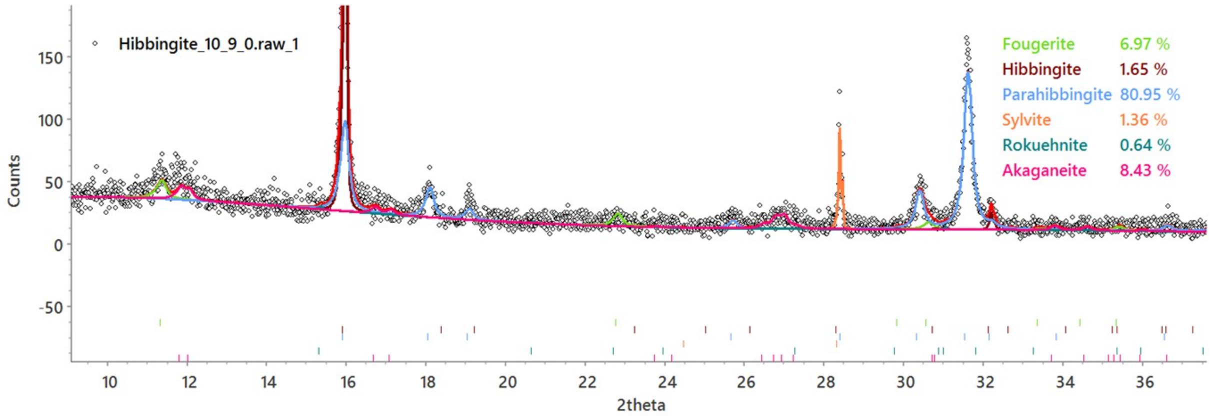

Sa

mple $Fe_2(OH)_3Cl(cr)$ 1.0 M KCl, pH 9

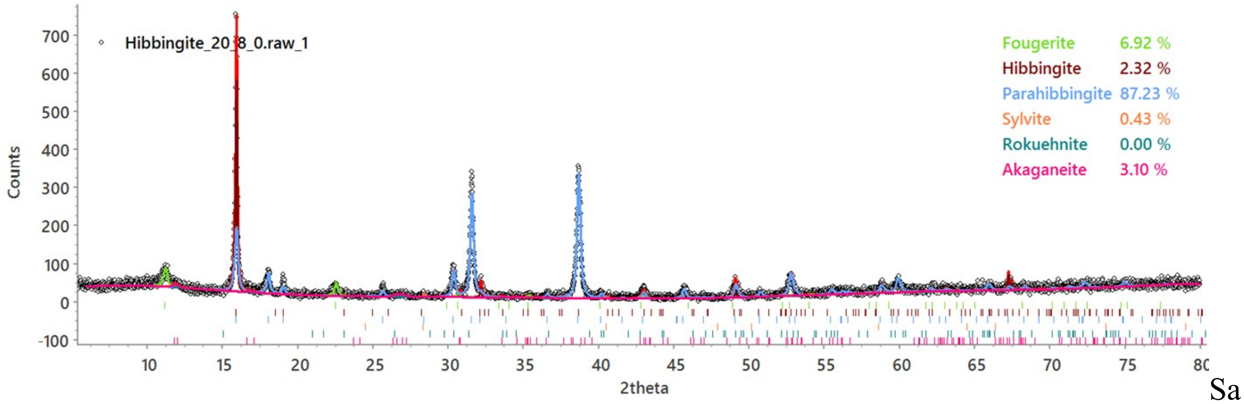

Sa

mple $Fe_2(OH)_3Cl(cr)$ 2.0 M KCl, pH 8

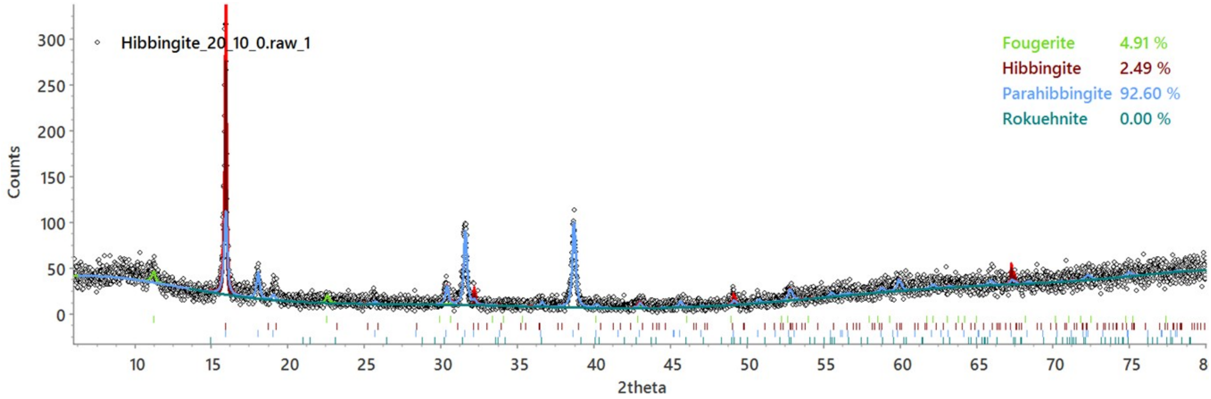

Sa

mple $Fe_2(OH)_3Cl(cr)$ 2.0 M KCl, pH 9

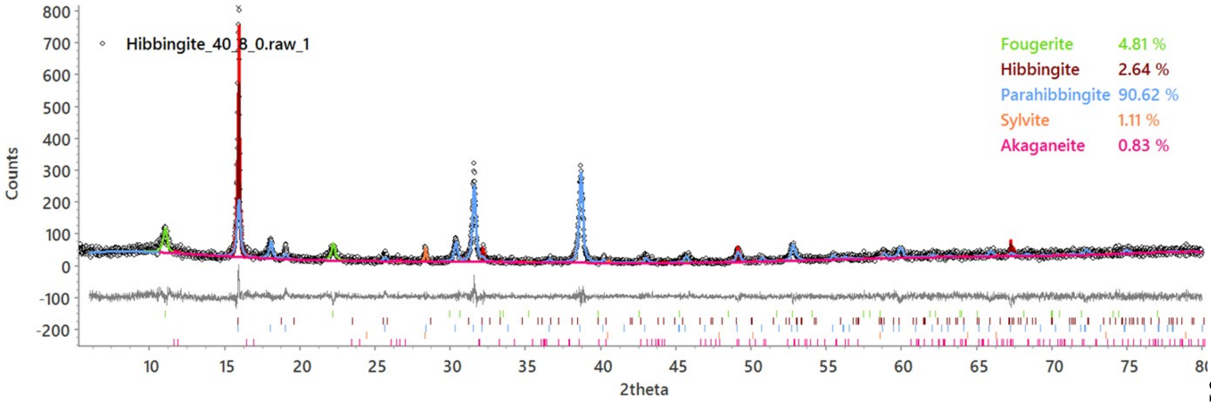

Sa

mple 4.0 M KCl, pH 8

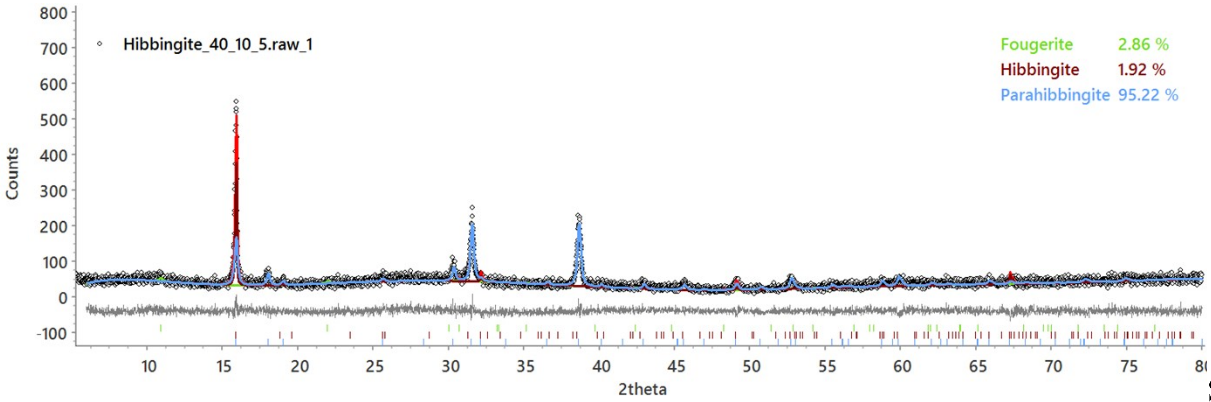

Sa

mple $Fe_2(OH)_3Cl(cr)$ 4.0 M KCl, pH 10.5

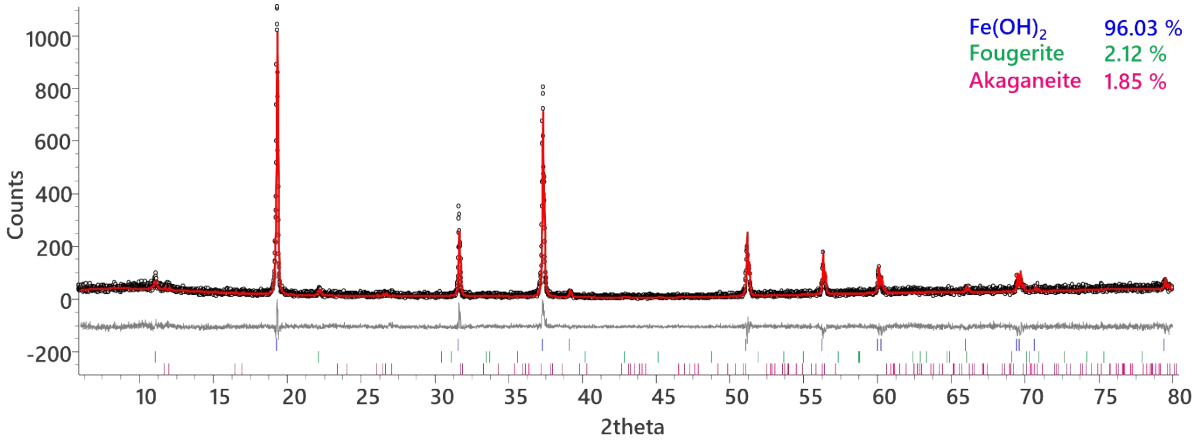

Sa

mple Mix M 0.5 KCl, pH 8

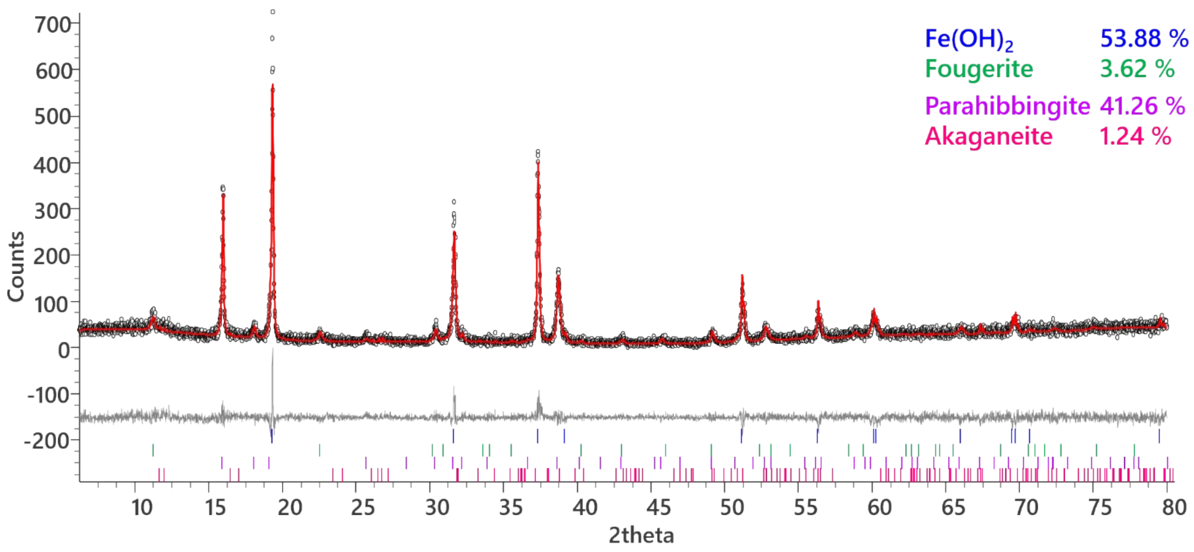

Sample Mix M 1.0 KCl, pH 8

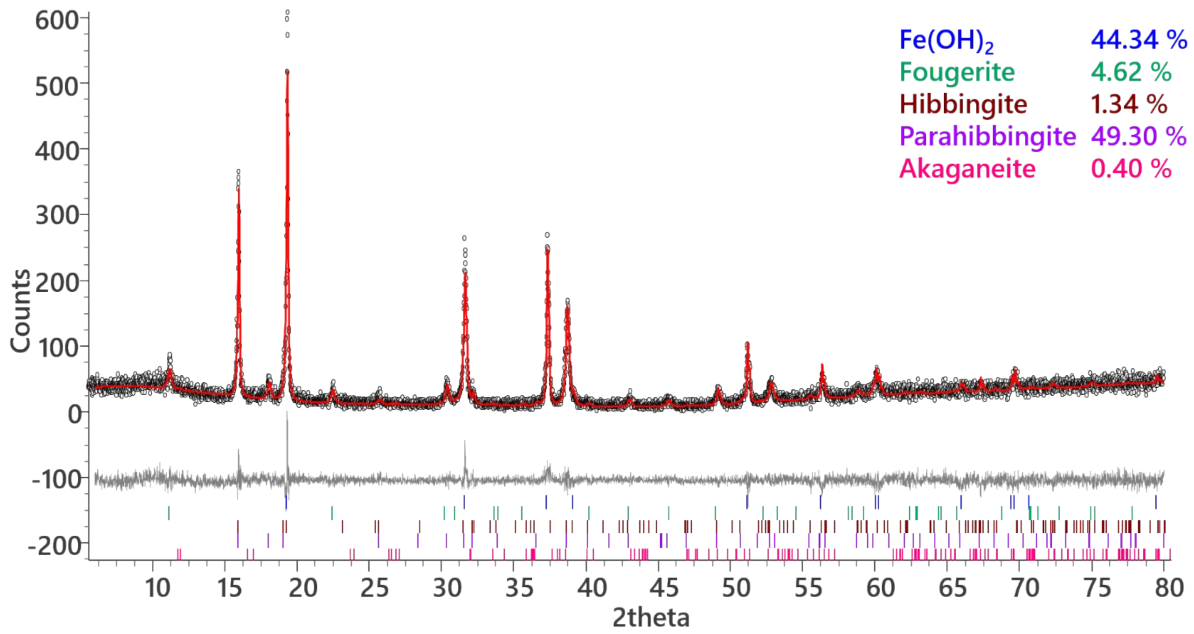

Sample Mix M 2.0 KCl, pH 8

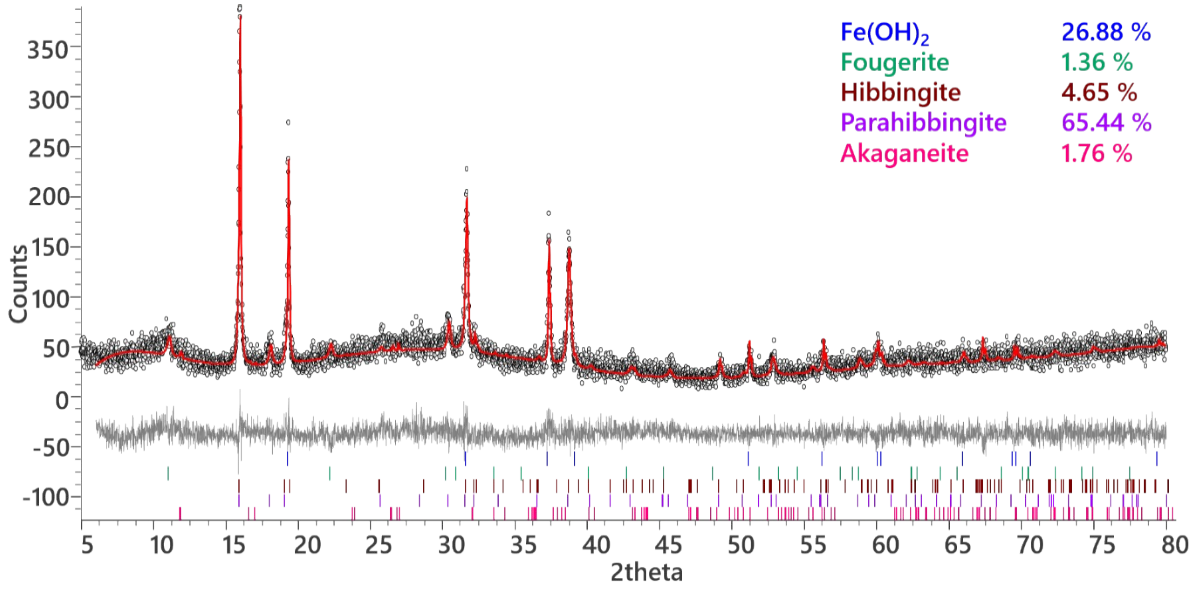

Sample Mix M 4.0 KCl, pH 8

Figure SI 2. *Rietveld plots of the investigated solid phases. Sample description below the corresponding figure.*

Table SI 2. *Results for $Fe(OH)_2$ samples from Rietveld refinements: crystallite size, unit cell parameters, criteria of fit. * Crystal size after Double Voigt approach.*

Sample	$Fe(OH)_2$ properties					Criteria of fit	
	Crystal size LVol-IB *	ucp. *a* (Å)	ucp. *c* (Å)	cell volume Å^3	PO**	GOF	DWS
$Fe(OH)_2$(cr)	132(2)nm	3.2683(2)	4.6021(4)	42.575(6)	0.55(1)	1.22	1.38
0.01 M KCl, pH_m 8.0	239(9)nm	3.2674(3)	4.6021(5)	42.548(9)	0.39(4)	1.13	1.67
0.01 M KCl, pH_m 10.1	177(4)nm	3.2675(3)	4.6016(5)	42.549(8)	0.57(2)	1.18	1.63
0.1 M KCl, pH_m 8.7	265(16)nm	3.2678(4)	4.6021(6)	42.56(1)	0.49(1)	1.05	1.86
0.1 M KCl, pH_m 10.0	254(10)nm	3.2681(3)	4.6023(5)	42.569(9)	0.20(3)	1.12	1.73
0.5 M KCl, pH_m 8.2	161(3)nm	3.2663(4)	4.6026(6)	42.525(12)	0.50(2)	1.19	1.5
0.5 M KCl, pH_m 9.5	194(6)nm	3.2688(2)	4.6030(3)	42.595(5)	0.546(5)	1.44	1.2
1.0 M KCl, pH_m 8.1	218(8)nm	3.2673(6)	4.6032(9)	42.556(17)	0.55(3)	1.12	1.74
1.0 M KCl, pH_m 9.5	320(20)nm	3.2700(4)	4.6029(5)	42.626(13)	0.26(5)	1.19	1.59
2.0 M KCl, pH_m 8.1	173(4)nm	3.2685(3)	4.6014(5)	42.572(10)	0.33(4)	1.25	1.39
2.0 M KCl, pH_m 9.5	239(8)nm	3.2682(4)	4.6018(5)	42.566(11)	0.30(6)	1.2	1.53
4.0 M KCl, pH_m 9.0	165(5)nm	3.2671(3)	4.6037(5)	42.555(10)	0.52(1)	1.22	1.43
4.0 M KCl, pH_m 9.4	219(9)nm	3.2673(4)	4.6051(6)	42.573(12)	0.42(1)	1.22	1.43

Table SI 3. *Results for $Fe_2(OH)_3Cl$ samples from Rietveld refinements: crystallite size, unit cell parameters, criteria of fit.*

Sample	β-$Fe_2(OH)_3Cl$ properties				Criteria of fit	
	Crystal size LVol-IB *	ucp. *a* (Å)	ucp. *c* (Å)	cell volume (Å^3)	GOF	DWS
$Fe_2(OH)_3Cl$ (cr)	32.2(5)	6.942(2)	14.739(4)	615.2(4)	1.20	1.44
0.5 M KCl, pH_m 7.6	31.8(5)	6.9414(1)	14.743(3)	614.8(3)	1.17	1.50
0.5 M KCl, pH_m 8.5	32(1)	6.934(4)	14.729(10)	613.3(9)	1.14	1.62
1.0 M KCl, pH_m 7.7	30.7(6)	6.939(3)	14.730(6)	614.3(5)	1.13	1.60
1.0 M KCl, pH_m 8.4	31.3(9)	6.939(2)	14.737(5)	614.6(4)	1.09	1.86
2.0 M KCl, pH_m 8.1	31.7(6)	6.9398(19)	14.736(4)	614.6(4)	1.14	1.65
2.0 M KCl, pH_m 9.4	33(1)	6.937(4)	14.742(9)	614.3(9)	1.09	1.79
4.0 M KCl, pH_m 8.1	31.1(6)	6.941(2)	14.736(5)	614.8(5)	1.13	1.65
4.0 M KCl, pH_m 9.7	31.3(10)	6.940(3)	14.739(7)	614.8(7)	1.08	1.77

Table SI 4. *Rietveld refinements conducted with XRD data after equilibration in 5 – 5.97 m NaCl kindly provided by Nemer et al.*[6]*: crystallite size and unit cell parameters.*

Sample	Properties			
	Crystal size LVol-IB *	ucp. *a* (Å)	ucp. *c* (Å)	cell volume (Å^3)
$Fe(OH)_2$(s) (Fig. 6 upper scan)	34.0(8)	3.2667(16)	4.604(2)	42.55(5)
$Fe_2(OH)_3Cl$(s) (Fig. 2 middle scan)	31.4(13)	6.925(8)	14.795(18)	614.5(16)

Table SI 5. *CE-ICP-MS results for selected samples from $Fe(OH)_2$(cr) undersaturation solubility experiments.*

Initial solid phase	*I* in M	pH_m	Share of Fe(II) in %
$Fe(OH)_2$(cr)	0.01	7.92 ± 0.24	91.1 ± 5.0
$Fe(OH)_2$(cr)	0.01	8.18 ± 0.17	98.1 ± 0.3
$Fe(OH)_2$(cr)	0.01	8.63 ± 0.11	89.2 ± 4.2
$Fe(OH)_2$(cr)	0.01	8.76 ± 0.07	90.1 ± 4.2
$Fe(OH)_2$(cr)	0.1	8.11 ± 0.26	91.44 ± 2.9
$Fe(OH)_2$(cr)	0.5	8.18 ± 0.14	93.8 ± 1.3
$Fe(OH)_2$(cr)	1.0	8.07 ± 0.09	95.6 ± 0.6
$Fe(OH)_2$(cr)	2.0	8.08 ± 0.15	95.4 ± 0.6
$Fe_2(OH)_3Cl$(cr)	0.5	7.64 ± 0.16	95.6 ± 1.5

$Fe_2(OH)_3Cl(cr)$	1.0	7.67 ± 0.04	96.0 ± 1.6
$Fe_2(OH)_3Cl(cr)$	2.0	8.06 ± 0.12	97.2 ± 1.6
$Fe_2(OH)_3Cl(cr)$	4.0	8.13 ± 0.19	97.3 ± 1.7
$Fe_2(OH)_3Cl(cr)$	0.5	8.47 ± 0.12	99.7 ± 1.0
$Fe_2(OH)_3Cl(cr)$	1.0	8.39 ± 0.14	98.6 ± 2.1

Table SI 6. *Results of solubility experiments given with one standard deviation. Mixed samples include both $Fe(OH)_2(cr)$ and $Fe_2(OH)_3Cl(cr)$ *Only one datapoint available. **Not included in model calculation, since no $Fe_2(OH)_3(cr)$ was present in the sample after equilibration.*

**Initial solid phase**	***I* in M**	**pH_m**	**log [Fe_{tot}]**
$Fe(OH)_2(cr)$	0.01	7.92 ± 0.24	−3.34 ± 0.02
$Fe(OH)_2(cr)$	0.01	8.18 ± 0.17	−4.04 ± 0.06
$Fe(OH)_2(cr)$	0.01	8.63 ± 0.11	−4.81 ± 0.03
$Fe(OH)_2(cr)$	0.01	8.76 ± 0.07	−5.16 ± 0.02
$Fe(OH)_2(cr)$	0.01	9.35 ± 0.21	−5.89 ± 0.28
$Fe(OH)_2(cr)$	0.01	10.10 ± 0.07	−6.96 ± 0.06
$Fe(OH)_2(cr)$	0.1	8.66 ± 0.15	−4.71 ± 0.07
$Fe(OH)_2(cr)$	0.1	8.11 ± 0.26	−3.63 ± 0.05
$Fe(OH)_2(cr)$	0.1	8.64 ± 0.14	−4.72 ± 0.11
$Fe(OH)_2(cr)$	0.1	9.55 ± 0.17	−6.04 ± 0.29
$Fe(OH)_2(cr)$	0.1	9.30 ± 0.14	−5.71 ± 0.28
$Fe(OH)_2(cr)$	0.1	9.95 ± 0.08	−6.55*
$Fe(OH)_2(cr)$	0.5	8.18 ± 0.14	−3.54 ± 0.02
$Fe(OH)_2(cr)$	0.5	8.90 ± 0.09	−4.82 ± 0.07
$Fe(OH)_2(cr)$	0.5	9.50 ± 0.09	−5.77 ± 0.09
$Fe(OH)_2(cr)$	1.0	8.07 ± 0.09	−3.36 ± 0.02
$Fe(OH)_2(cr)$	1.0	8.90 ± 0.09	−4.86 ± 0.06
$Fe(OH)_2(cr)$	1.0	9.46 ± 0.12	−5.77 ± 0.11
$Fe(OH)_2(cr)$	2.0	8.08 ± 0.15	−3.41 ± 0.03
$Fe(OH)_2(cr)$	2.0	8.97 ± 0.12	−4.91 ± 0.18

$Fe(OH)_2(cr)$	2.0	9.47 ± 0.15	−5.55 ± 0.08
$Fe(OH)_2(cr)$	4.0	9.04 ± 0.18	−4.67 ± 0.06
$Fe(OH)_2(cr)$	4.0	9.04 ± 0.13	−4.63 ± 0.04
$Fe(OH)_2(cr)$	4.0	9.43 ± 0.19	−5.19 ± 0.05
$Fe_2(OH)_3Cl(cr)$	0.5	7.64 ± 0.16	−2.59 ± 0.03
$Fe_2(OH)_3Cl(cr)$	0.5	8.06 ± 0.06	−3.36 ± 0.03
$Fe_2(OH)_3Cl(cr)$	0.5	8.47 ± 0.12	−4.06 ± 0.15
$Fe_2(OH)_3Cl(cr)$	1.0	7.67 ± 0.04	−2.65 ± 0.03
$Fe_2(OH)_3Cl(cr)$	1.0	8.01 ± 0.15	−3.38 ± 0.04
$Fe_2(OH)_3Cl(cr)$	1.0	8.39 ± 0.14	−4.08 ± 0.20
$Fe_2(OH)_3Cl(cr)$	2.0	8.06 ± 0.12	−3.32 ± 0.03
$Fe_2(OH)_3Cl(cr)$	2.0	8.24 ± 0.16	−3.50 ± 0.07
$Fe_2(OH)_3Cl(cr)$	2.0	8.59 ± 0.18	−4.21 ± 0.22
$Fe_2(OH)_3Cl(cr)$	2.0	8.99 ± 0.13	−4.66 ± 0.23
$Fe_2(OH)_3Cl(cr)$	2.0	9.38 ± 0.19	−5.15 ± 0.32
$Fe_2(OH)_3Cl(cr)$	4.0	8.13 ± 0.19	−2.50 ± 0.03
$Fe_2(OH)_3Cl(cr)$	4.0	8.43 ± 0.12	−3.36 ± 0.04
$Fe_2(OH)_3Cl(cr)$	4.0	8.66 ± 0.17	−3.92 ± 0.13
$Fe_2(OH)_3Cl(cr)$	4.0	8.94 ± 0.17	−4.37 ± 0.45
$Fe_2(OH)_3Cl(cr)$	4.0	9.29 ± 0.22	−4.82 ± 0.25
$Fe_2(OH)_3Cl(cr)$	4.0	9.66 ± 0.26	−5.28 ± 0.58
Mixed**	0.5	7.88 ± 0.27	−3.04 ± 0.14
Mixed	1.0	8.04 ± 0.15	−3.30 ± 0.10
Mixed	2.0	8.53 ± 0.07	−3.86 ± 0.13
Mixed	4.0	9.11 ± 0.10	−4.34 ± 0.04

Table SI 7. *SIT interaction coefficients used in model development and determined within this study.*

**Ion interaction coefficient**	**Value in kg·mol^{-1}**	**Reference**
ε(H^+, Cl^-)	0.12 ± 0.01	Reported by Ciavatta[23]
ε(K^+, Cl^-)	0 ± 0.01	Reported by Ciavatta[23]
ε(Fe^{2+}, Cl^-)	0.17 ± 0.01	Selected in NEA-TDB[2]
ε($Fe(OH)^+$, Cl^-)	0.05 ± 0.1	Estimated based on Hummel[42]
ε($Fe(OH)_2$, Cl^-)	0	By definition in SIT
ε($FeCl^+$, Cl^-)	0.16 ± 0.01	Discussed in NEA-TDB[2]

Table SI 8. *Conditional solubility constants log *K'$_{s,0}$($Fe_2(OH)_3Cl$(cr)) derived from the samples of the mixed systems where both phases were still present after equilibration given with two standard deviations of the averaged data from three samplings as uncertainty.*

**Ionic strength in M**	**log *K'$_{s,0}$($Fe_2(OH)_3Cl$(cr))**
1.0	17.66 ± 0.31
2.0	17.82 ± 0.14
4.0	18.17 ± 0.20

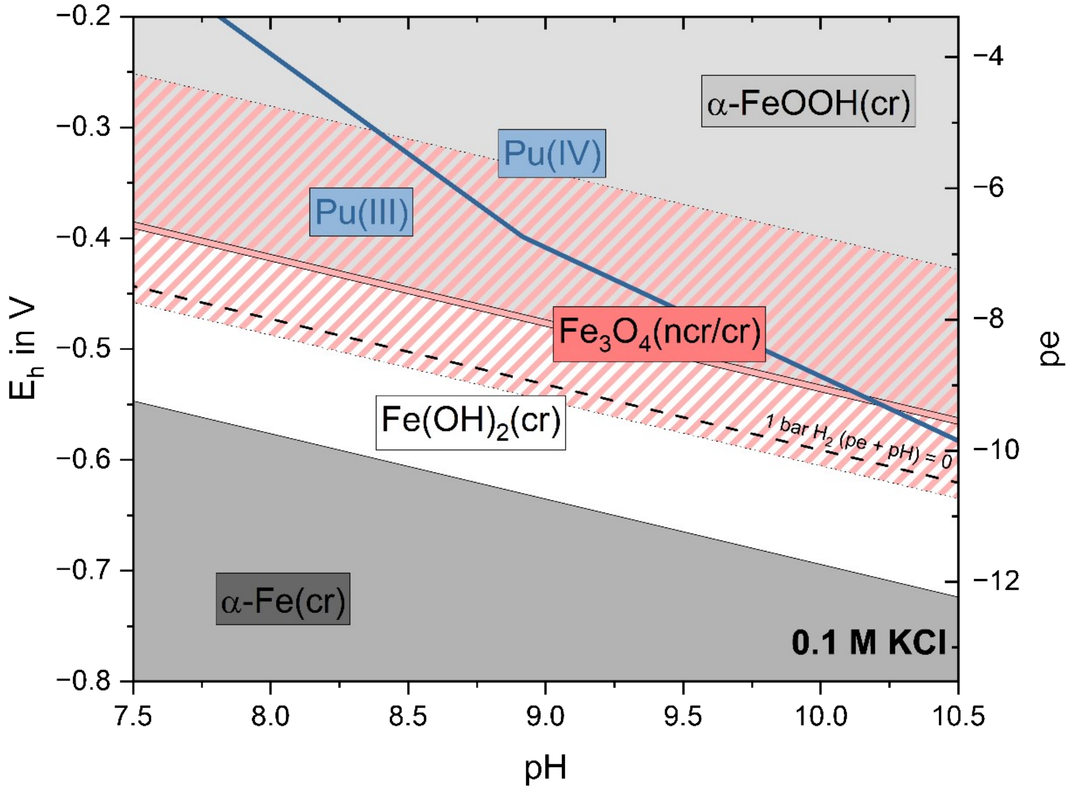

Figure SI 3. *Modified Figure 7 including the redox borderline in the aqueous system between Pu(III) and Pu(IV) calculated using data in ThermoChimie.*[10]
